# Salinomycin as a death switch: how gastric cancer cells choose their demise

**DOI:** 10.1038/s41420-026-03058-2

**Published:** 2026-03-24

**Authors:** Pasqualina Laurenziello, Margherita Luongo, Francesca Lospinoso Severini, Giovanni Calice, Ottavia Bartolo, Geppino Falco, Carlo Calabrese, Sabino Russi, Simona Laurino

**Affiliations:** 1 IRCCS CROB Centro di Riferimento Oncologico della Basilicata, Rionero in Vulture, Italy; 2 Department of Biology, University of Naples Federico II, Naples, Italy; 3 Biogem, Istituto di Biologia e Genetica Molecolare, Ariano Irpino, Italy

**Keywords:** Preclinical research, Gastric cancer

## Abstract

Gastric cancer (GC) remains a significant global health challenge due to the prevalence of multidrug resistance (MDR) that leads to therapy failure. MDR is driven by tumor heterogeneity and the presence of cancer stem cells (CSCs). Drug repurposing represents an innovative therapeutic strategy to overcome MDR. In this view, Salinomycin (Sal) has shown promising anticancer activity and selectivity against CSCs. Since its mechanisms in GC are not fully understood, we investigated its activity in a panel of four GC cell lines: SNU1, NCI-N87, AGS, and KATO-III. Our results demonstrate that Sal induces distinct forms of regulated cell death (RCD) in a cell line-specific manner. Sal treatment led to apoptosis in SNU1 and NCI-N87 cells, while it triggered ferroptosis in AGS and KATO-III cells. Autophagy was a common early event in all cell lines. Western blot analysis confirmed the activation of distinct signaling axes: mTOR/survivin/CASP-3/BAX in apoptotic cells and mTOR/survivin/SLC7A11/GPX4 in ferroptotic cells. Bioinformatics analysis revealed a unique 20-differentially expressed gene signature for ferroptosis-prone GC cells. Notably, Sal significantly reduced the proportion of CD44^+^ and CD133^+^ CSCs in the drug-resistant KATO-III and NCI-N87 cell lines. By selectively inducing either apoptosis or ferroptosis, Sal effectively overcomes MDR and targets the CSC population by reducing the capability to form spheroids and colonies. Moreover, our ferroptosis-related gene signature resulted useful to stratify GC patients and was found associated with better outcomes, highlighting the translational potential of Sal treatment. Indeed, it was effective to promote both apoptotic and ferroptotic RCD on patient-derived gastric cancer organoids. Notably, autophagy was a common RCD mechanism also in this preclinical model. Our findings suggest that Sal is a promising candidate for GC treatment, and understanding a tumor’s specific molecular susceptibilities could enable the development of personalized therapeutic strategies.

## Introduction

Gastric Cancer (GC) is one of the most frequent and aggressive tumors worldwide, characterized by high mortality rates [1]. Its clinical management is complicated by late-stage diagnosis, rapid progression, and poor efficacy of current treatment options [2]. Multidrug resistance (MDR) is a leading cause of treatment failure and disease relapse [3]. Two major biological determinants of MDR include tumor heterogeneity and the presence of Cancer Stem Cells (CSCs). On one hand, tumor heterogeneity creates a diverse landscape of cell populations with different sensitivities to chemotherapy. On the other hand, conventional cancer therapies are designed to target the rapidly proliferating tumor bulk, failing to eradicate the quiescent or slow-cycling CSCs [4]. To overcome the interplay between tumor bulk heterogeneity and resistant CSC, and to achieve lasting therapeutic success, innovative strategies that can simultaneously target these two cancer features are needed.

In this view, repurposing of existing drugs with recognized anticancer activity represents a valid strategy to improve GC clinical management. Salinomycin (Sal), a polyether ionophore originally used in veterinary medicine, has emerged as a promising anticancer agent also selective against CSCs [5]. Sal induces cell death through multiple, distinct, and interconnected pathways. Recent literature report that its action is not limited to a single mechanism but represents a multi-pronged assault on cellular survival, encompassing apoptosis, autophagy, and a unique form of non-apoptotic death known as ferroptosis [6–9]. Although apoptosis and autophagy represent well-characterized and widely studied forms of regulated cell death (RCD) [10], ferroptosis, a distinct iron-dependent mechanism with unique biochemical features [11], has gained attention as a promising strategy to overcome resistance to conventional treatments. In particular, unlike apoptosis, which relies on caspase activation, and autophagy, which involves lysosomal degradation pathways, ferroptosis is driven by lipid peroxidation and dysregulated redox homeostasis. Understanding the molecular mechanisms underlying and triggering this specific type of cell death is therefore crucial, as it could significantly enhance the effectiveness of current therapies and pave the way for innovative treatment strategies [12, 13]. However, the precise mechanisms by which Sal exerts its anti-tumor effects in GC [14], also in relation to CSCs, remain to be understood.

Here, we demonstrated the ability of Sal to trigger autophagy, apoptosis and ferroptosis in GC cell lines. Specifically, we outlined in depth the molecular pathways engaged, exploring the interplay between these different cell death mechanisms and identifying a signature of key molecular regulators that dictate the balance between them. Sal, through its induction of oxidative stress, triggers a coordinated cascade of cell death pathways, with ferroptosis playing a previously uncharacterized but significant role in GC. This research will contribute to a more comprehensive understanding of Sal-induced regulated cell death (RCD) mechanisms able to overcome MDR by targeting both tumor bulk and therapy-resistant CSC, making it a highly promising candidate for GC treatment.

## Results

### Salinomycin reduces viability of gastric cancer cell lines

Aimed to evaluate the anti-tumor activity of Sal in GC, we employed a panel of four GC cell lines (SNU1, NCI-N87, AGS, and KATO-III). We performed a dose-response assay to estimate the IC50 values, finding cell line- and time-dependent differences in treatment response. As shown in Supplementary Fig. S1 and Supplementary Table S1, AGS and SNU1 were the more sensitive to Sal as compared to NCI-N87 and KATO-III. Considering the doubling times and that IC50 estimation was statistically significant at 48 h for all cell lines, we used this time point and the relative IC50 values to perform further experiments.

### Salinomycin promotes apoptosis, necrosis, and ferroptosis in a cell line-specific manner

**Fig. 1:**
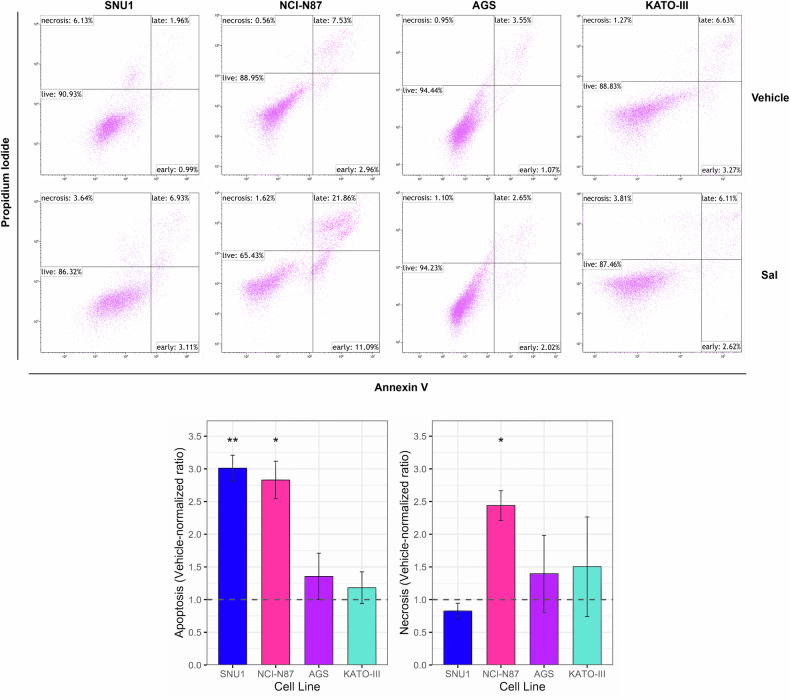
Salinomycin treatment promoted apoptotic cell death. Scatter plots following 48 h of Sal treatment of different GC cell lines analyzed by flow cytometry, using Annexin V-FITC and PI labeling. Representative dot plots present the percentage of live cells in the lower-left quadrant, early apoptotic cells in the lower-right quadrant, late apoptotic cells in the upper-right quadrant and necrotic cells in the upper-left quadrant. Histogram reports frequencies of apoptotic and necrotic cells as ratios of Sal-treated to vehicle for each tested cell line. Sal significantly promoted apoptosis in two out of 4 cell lines. Data were obtained from three independent biological replicates. One-sample *t*-test was used to evaluate statistical significance (*p < 0.05; **p < 0.01).

As first cell death mechanisms, we evaluated apoptosis activation by flow cytometry using the Annexin V/Propidium Iodide assay. Each cell line was treated for 48 h at the estimated IC50 (SNU1: 6 µM; NCI-N87: 12 µM; AGS: 6 µM; KATO-III: 18 µM), equivalent volumes of Sal vehicle (DMSO) were used as controls. Interestingly, a marked increase in the rate of apoptotic cells following Sal treatment was observed only in SNU1 and NCI-N87 (15.9 ± 3.3 vs 5.4 ± 1.3, *p* = 0.009; 39.8 ± 3.5 vs 14.6 ± 2.7, *p* = 0.02). The latter also showed an induction of necrotic cell death (2.6 ± 0.5 vs 1.1 ± 0.3, *p* = 0.02). Conversely, the viability reduction observed for AGS and KATO-III cells was not associated with these cell death mechanisms (Fig. 1; Supplementary Table S2).

**Fig. 2:**
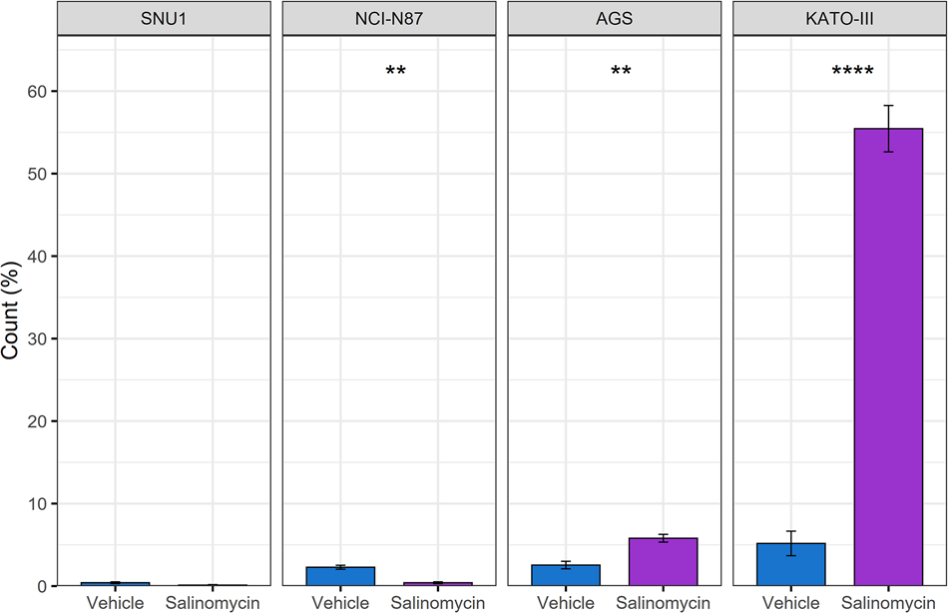
Salinomycin treatment promoted ROS production in GC cell lines. H2DCFA was used to evaluate ROS production after Sal treatment (48 h) by flow cytometry. Histogram reports the frequencies of ROS positive cells. Sal significantly promoted ROS production in the two cell lines (AGS and KATO-III) not undergoing apoptotic cell death. Data were obtained from three independent biological replicates. T-test was used to evaluate statistical significance (**p < 0.01, ****p < 0.0001).

**Fig. 3:**
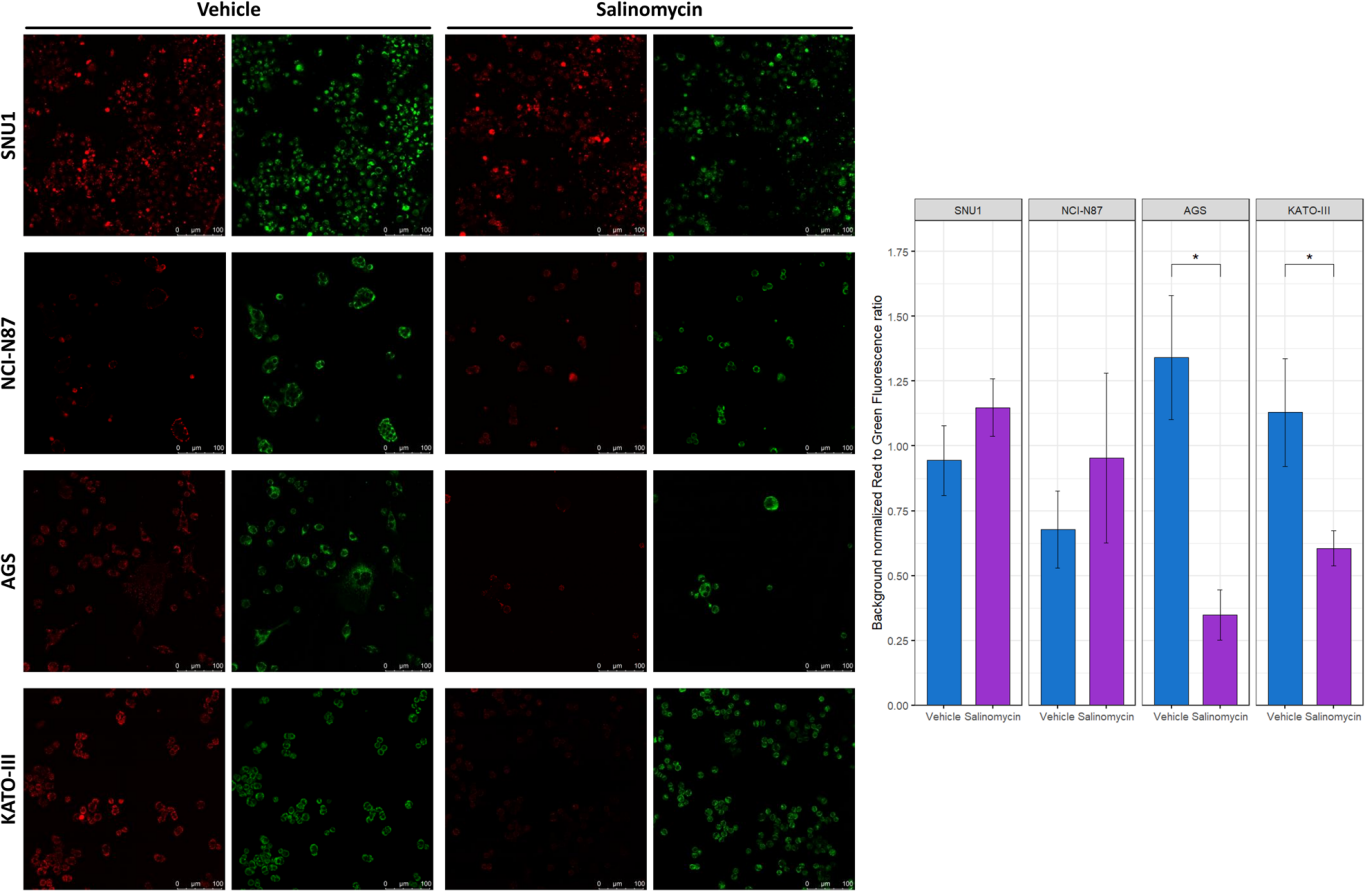
Salinomycin treatment led to mitochondrial membrane depolarization. Cells were treated with Sal for 48 h and then stained with JC-1 fluorescent probe (10 µM) to assess alteration of mitochondria membrane potential by evaluating the red (aggregates in healthy mitochondria) to green (monomers in unhealthy mitochondria) fluorescence shift. After live cells observation (Magnification 20×), the red to green fluorescence intensities ratio was estimated. A significant reduction in this ratio, as compared with vehicle-treated cells, was recorded for ROS producing and not for apoptotic-prone cell lines. Data were obtained from three independent biological replicates. *T*-test was used to evaluate statistical significance (*p < 0.05).

Since Sal was found to induce ferroptosis in other cancers, we further investigated in GC this distinct RCD in which the buildup of ROS is a trigger. Using H2DCFDA, we found that Sal elicited an increase in intracellular ROS only in AGS (5.8 ± 0.5 vs 2.5 ± 0.5, *p* = 0.001) and KATO-III (55.4 ± 2.8 vs 5.2 ± 1.5, *p* = 9.3 ×10^−5^) cells. Conversely, lower rates of ROS positive cells were observed for SNU1 (0.1 ± 0.1 vs 0.4 ± 0.1, *p* = 0.07) and NCI-N87 (0.4 ± 0.1 vs 2.3 ± 0.2, *p* = 0.002) cell lines after exposure to Sal (Fig. 2). Since it is known that loss of MMP is a ferroptosis hallmark, we used JC-1 staining to evaluate Sal activity on this organelle. By estimating the alteration of red (high MMP, healthy) to green (low MMP, unhealthy) fluorescence ratio, we found a significant mitochondrial membrane depolarization (lower ratios) following Sal treatment in AGS (0.3 ± 0.1 vs 1.3 ± 0.2, *p* = 0.02) and KATO-III (0.6 ± 0.1 vs 1.1 ± 0.2, *p* = 0.04) cells (Fig. 3), supporting the activation of ferroptosis. No significant alterations of MMP were observed in SNU1 (1.1 ± 0.1 vs 0.9 ± 0.1, *p* = 0.3) and NCI-N87 (0.9 ± 0.3 vs 0.7 ± 0.1, *p* = 0.6) cells.

### Autophagy is an early trigger of Sal-induced apoptotic and ferroptotic cell death

**Fig. 4:**
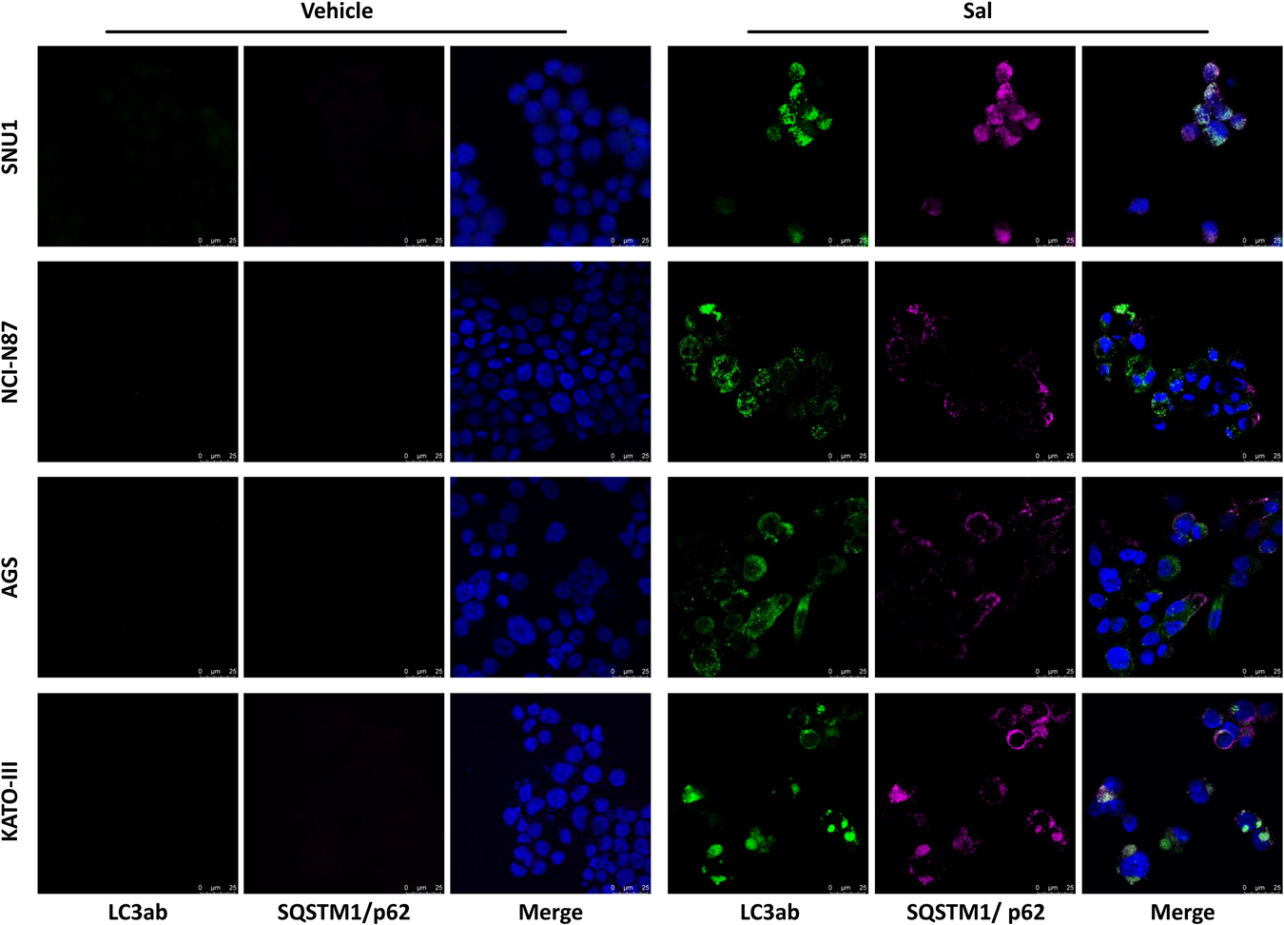
Autophagy was an early RCD common to all GC cell lines. Cells were treated with Sal for 48 h, detecting LC3A/B and SQSTM1/p62 expression and localization by immunofluorescence. Increase of puncta formation for both markers supported the involvement of autophagy in all cell lines (blue: nuclei, green: LC3A/B, magenta: SQSTM1/p62; Magnification 20×).

**Fig. 5:**
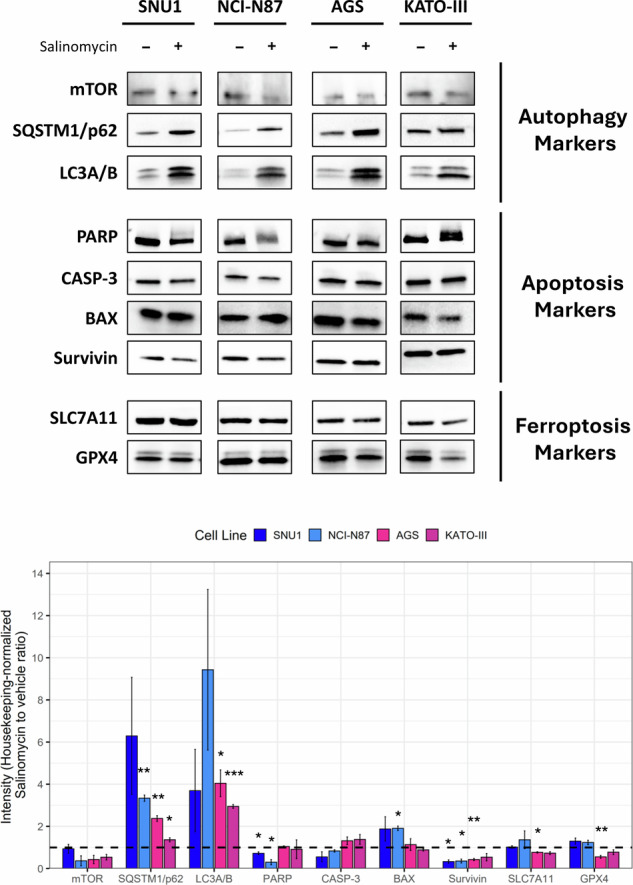
Salinomycin promoted GC cell line RCD through different signaling pathways. Expression of specific autophagy, apoptosis, and ferroptosis markers was assessed by western blot. Results outlined a common activation of autophagy that induced a ferroptotic or apoptotic RCD through the modulation of mTOR/survivin/SLC7A11/GPX4 or mTOR/survivin/CASP-3/BAX pathways, respectively. Densitometry from three independent experiments was reported as histogram. One-sample t-test was used to evaluate statistical significance (*p < 0.05, **p < 0.01, ***p < 0.001). Housekeeping genes used for normalization were ACTB, GAPDH, α/β-Tubulin, and vinculin, according to molecular weight of target proteins on the blot.

Since several works indicate autophagy as a gatekeeper of cell fate modulating both apoptosis and ferroptosis, we investigated this additional RCD. Notably, in all cell lines, immunofluorescence showed an upregulation of recognized autophagy markers, SQSTM1/p62 and LC3A/B, in Sal-treated cells as compared with controls (Fig. 4). This finding confirmed its activation and suggested that it could be a common cell clearance mechanism triggering different cell death mechanisms. To elucidate the signaling axes influencing the fate of GC cells following Sal treatment, we measured protein levels of several key regulators of these cell death mechanisms by western blot. Interestingly, the activation of autophagy was also supported by the down-regulation of mTOR. Moreover, we observed reduced levels of PARP and CASP-3 apoptosis activation markers zymogen forms following Sal treatment only in SNU1 and NCI-N87 cells. We also found the upregulation of pro-apoptotic protein BAX and down-regulation of survivin, an inhibitor of apoptosis. Furthermore, we demonstrated a down-regulation of SLC7A11 and GPX4 in ferroptosis-prone cell lines. Overall, our results highlighted two RCD signaling axes characteristic of ferroptosis- and apoptosis-prone cell lines: mTOR/survivin/SLC7A11/GPX4 and mTOR/survivin/CASP-3/BAX, respectively (Fig. 5).

### The final decision between ferroptotic or apoptotic RCD reflects the cell’s molecular features

**Fig. 6:**
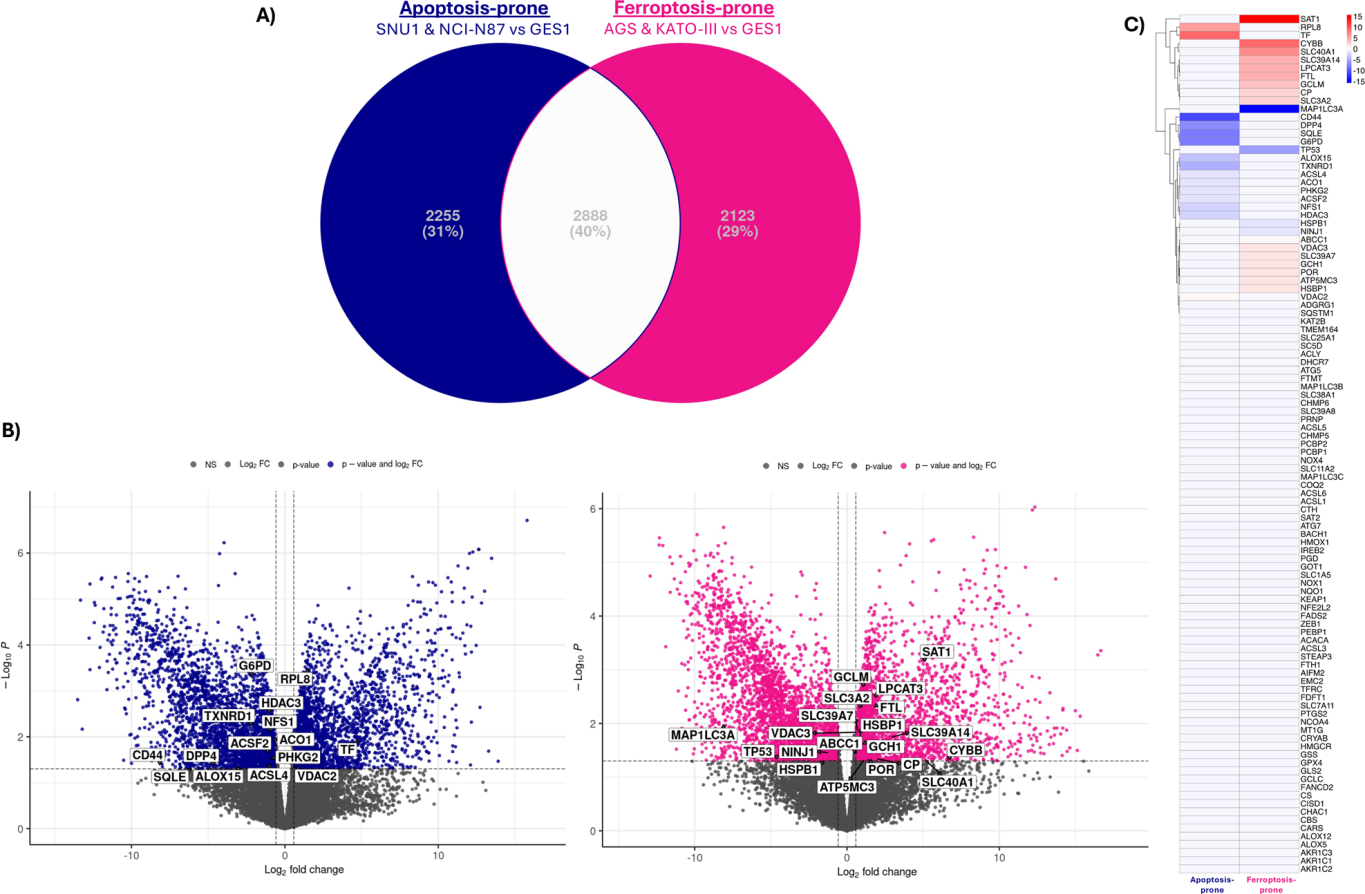
A ferroptosis-related gene signature characterizes ferroptosis-prone GC cell lines. Gene expression data of the four GC cell lines and gastric primary normal one (GES-1) were retrieved from public repositories. **A** In Venn diagram, differentially expressed genes (DEGs) numbers from comparisons of apoptosis- or ferroptosis-prone cell lines against normal stomach epithelial cell were reported (blue and pink, respectively). DEGs were filtered using *p* < 0.05. **B** DEGs, after excluding common genes between the two comparisons (white in Venn), were depicted in Volcano plots highlighting those matching with the three ferroptosis gene sets. **C** The heatmap highlights the 20 DEGs that defined the ferroptosis-related gene signature. DEGs were filtered for *p* < 0.05 and |log_2_FC| > 0.58 and reported as weighted Fold Change (logFC * -log10(*p* value)).

Aimed to decipher the molecular determinants of cell’s fate under Sal treatment, we performed a bioinformatics analysis to define a signature of ferroptosis-related genes in GC. We compared gene expression levels of the ferroptosis- and apoptosis-prone cell lines with the gastric primary normal one GES-1. The two lists of DEGs were matched to exclude common DEGs (*n* = 2888), obtaining two specific lists of DEGs (apoptosis-prone: *n* = 2255 and ferroptosis-prone: *n* = 2123) (Fig. 6A, B, Supplementary File S1). Moreover, we retrieved three lists of ferroptosis-related genes, one by Hong et al. (*n* = 60) [15], and two from MSigDB, the GOBP_FERROPTOSIS (*n* = 18) and the WP_FERROPTOSIS (*n* = 64). Notably, we identified for the first time a gene signature characteristic of ferroptosis-prone GC cell line. We found up-regulated ATP5MC3, GCLM, LPCAT3, SAT1, HSBP1, ABCC1, CP, CYBB, SLC39A14, FTL, GCH1, SLC40A1, POR, SLC3A2, VDAC3, SLC39A7 and down-regulated HSPB1, TP53, MAP1LC3A, NINJ1. These 20 DEGs defined a distinctive signature of GC cell lines undergoing ferroptosis RCD under Sal treatment. Conversely, in GC cell lines undergoing apoptosis, we found a down-regulation of several ferroptosis-related genes (ACSL4, ALOX15, CD44, DPP4, PHKG2, ACO1, NFS1, SQLE, G6PD, ACSF2, TXNRD1, HDAC3) (Fig. 6C, Supplementary File S1).

### Salinomycin is effective to overcome CSC-mediated drug resistance

**Fig. 7:**
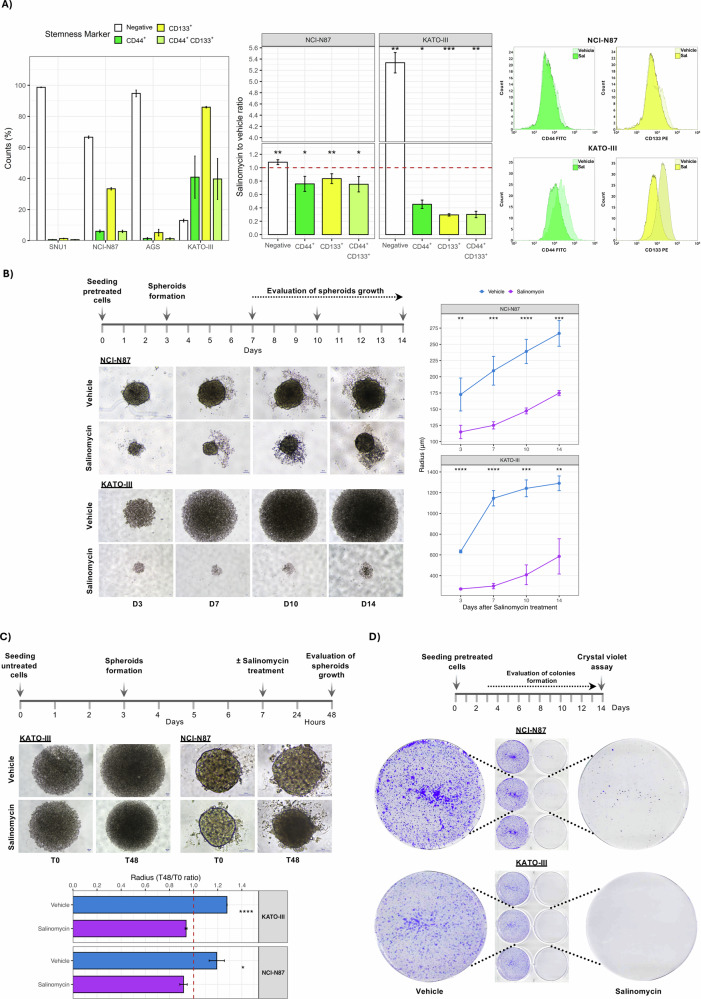
Salinomycin affects GC stemness markers CD44^+^ and CD133^+^ fractions. **A** Single cell suspension of Sal- and vehicle-treated cell lines were stained with anti-CD44-FITC and anti-CD133-PE and analyzed by flow cytometry. High levels of both CSC markers were measured in NCI-N87 and KATO-III cells with the latter showing the highest expression. These cell lines, after 48 h of Sal treatment, showed a marked reduction of CD44^+^ and CD133^+^ cell populations. Representative histogram overlays of Sal *vs* vehicle stemness markers positive cell populations. One-sample *t*-test was used to assess significance, using data from three independent biological replicates. **B** After 48 h of treatment, cells were harvested and seeded and cultured for 14 days. Morphology and size of spheroids were recorded at 3, 7, 10, and 14 days. The plots summarize radius of Sal-treated cells as compared with vehicle controls from three replicates of two independent experiments. Images were acquired at 10× and 4× for NCI-N87 and KATO-III, respectively. A marked reduction of spheroids size was observed for NCI-N87 cells, while no spheroids formation was observed for KATO-III. **C** Untreated cells were seeded and treated on day 7 after spheroid formation. Their morphology and size were assessed after 48 h of treatment. Plots report the ratio between spheroids radius before and after treatment from three independent experiments. Sal-treated spheroids were significantly smaller in size as compared with vehicle for both cell lines. **D** Cells were treated for 48 h and then harvested and seeded for colony-forming assay. Colonies were observed after 14 days of culture, few colonies formed in Sal-treated NCI-N87 cells, and no colonies were found for KATO-III cells. Three independent experiments were performed. *T*-test was employed to estimate significance (*p < 0.05, **p < 0.01, ***p < 0.001, ****p < 0.0001).

Salinomycin was proven to be highly effective against CSC. Flow cytometry analysis was used to assess the fraction of cells positive to the well-known GC-CSC markers, CD44 and CD133 [16]. According to our previous evidence about their lower sensitivity to cisplatin [17, 18], NCI-N87 and KATO-III cells showed a high percentage of cells positive for these two markers (Fig. 7A), sustaining a role for CSC in drug resistance. Specifically, KATO-III cell line was characterized by 86% CD133^+^ and 41% CD44^+^ cells, with about 40% of cells co-expressing both markers. This suggests that CD133 expression could drive that of CD44. Similarly, in the NCI-87 cell line, 33% of cells were CD133^+^ and 6% were CD44^+^. In contrast, the remaining two cell lines were nearly negative for these GC-CSC markers (CD133: 1% to 5%, CD44: 0% to 1%) (Supplementary Table S3). Notably, in both cell lines, Sal treatment led to a significant decrease in the proportion of CD44^+^, CD133^+^, and CD44^+^CD133^+^ cells. This was accompanied by a corresponding increase in double-negative cells fraction, with the most substantial change observed in the KATO-III cell line (Fig. 7A).

To further investigate the effectiveness of Sal against the CSC compartment, 3D cultures were employed. Indeed, several studies reported that cancer cell-derived spheroids are enriched in CSCs or cells expressing stemness markers [19]. Figure 7B shows that Sal treatment impairs the ability to form spheroids. This effect was more evident in KATO-III cell line characterized by a higher fraction of CD133 and CD44 positive cells. The line graph highlights the significant reduction of spheroids size at different observation time points. In addition, we performed a spheroid formation assay treating with Sal for 48 h spheroids obtained after 7 days in the appropriate culture medium. We observed a marked alteration of morphology and a smaller size in Sal-treated spheroids (Fig. 7C). We also evaluated the effect of Sal on colony formation capability by seeding pretreated cells. After 14 days, a dramatic reduction of colonies formation was recorded for NCI-N87 cell line, whereas no colonies were observed for KATO-III cell line as compared with vehicle-treated cells (Fig. 7D). These results sustain Sal as a promising candidate for overcoming drug resistance, a major challenge in cancer treatment, by its ability to target GC-CSC.

### Ferroptosis-related gene (FRG) signature is associated with molecular subtypes and survival outcomes in TCGA and ACRG gastric cancer patients cohorts

**Fig. 8:**
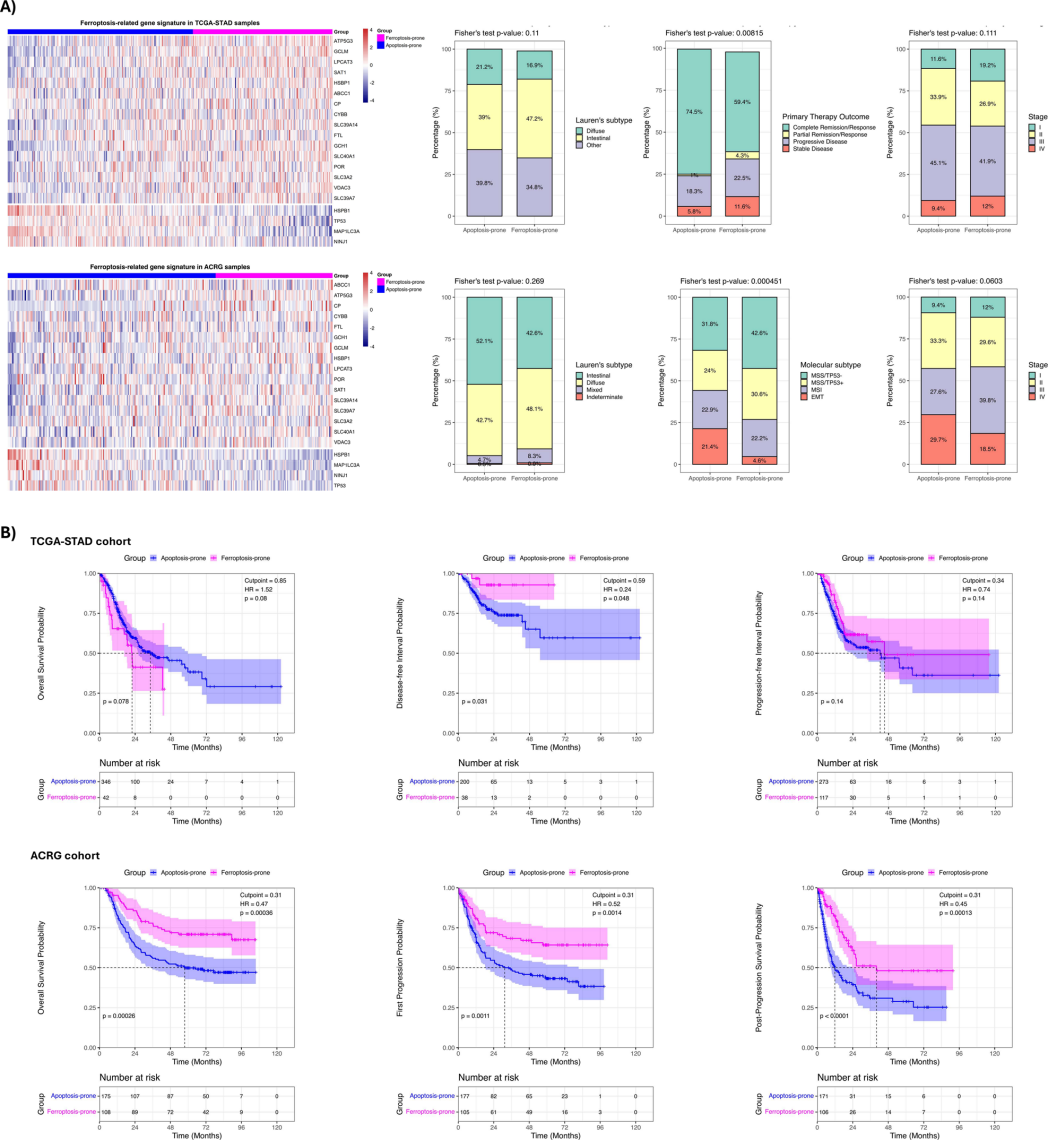
Association of ferroptosis-related gene (FRG) signature with clinicopathological features and outcomes in gastric cancer patients. **A** Gene expression data from TCGA-STAD (*n* = 414) and ACRG (*n* = 300) were downloaded to estimate the FRG SignatureScore, which was used to generate the heatmaps. FRG signature was able to classify patients in ferroptosis-prone and apoptosis-prone. Association of these subgroups with clinicopathological features was assessed. **B** Different outcomes data were used to evaluate prognostic value of the FRG signature, which was found to be associated with better progression- and relapse-related ones.

Across both TCGA-STAD and ACRG gastric cancer cohorts, patients were stratified by FRG SignatureScore into Ferroptosis-prone and Apoptosis-prone groups. In TCGA-STAD, the optimal cutoff identified 178 Ferroptosis-prone and 236 Apoptosis-prone patients, while in ACRG there were 108 Ferroptosis-prone and 192 Apoptosis-prone patients (Fig. 8A). In both cohorts, FRG SignatureScore showed limited associations with traditional clinicopathological features. In TCGA-STAD, Lauren’s subtypes (overall Fisher’s *p* = 0.11) and AJCC pathological stage (overall Fisher’s *p* = 0.111) were not significantly associated with FRG SignatureScore. On the contrary, among primary therapy outcomes, Complete Remission/Response was associated with low FRG SignatureScore (overall Fisher’s *p* = 0.008; Odds Ratio = 1.99; Holm’s *p*.adj = 0.022). Similarly, in ACRG cohort, Lauren’s subtypes (overall Fisher’s *p* = 0.27) and AJCC stage distributions (overall Fisher’s *p* = 0.06) showed no significant association with FRG signature expression. However, we observed a trend towards a higher proportion of Apoptosis-prone patients in Stage IV compared to the Ferroptosis-prone group (29.7% vs 18.5%, *p* = 0.01, Holm’s *p*.adj = 0.07). By contrast, molecular subtypes in ACRG were significantly associated with FRG SignatureScore (overall Fisher’s *p* = 0.0004). The most striking difference was observed in the EMT subtype, which was significantly less frequent in the Ferroptosis-prone group (4.63%) compared to the Apoptosis-prone group (21.35%) (MSS/TP53- vs EMT, Holm’s *p*.adj < 0.001). Conversely, the Ferroptosis-prone phenotype was predominantly enriched in the MSS/TP53- subtype (42.59%), suggesting a specific molecular background for this group. Despite the limited associations with clinicopathological features, FRG SignatureScore was prognostic in both cohorts (Fig. 8B). In TCGA-STAD, higher FRG SignatureScore (Ferroptosis-prone patients) tended to be associated with worse OS (HR = 1.52, 95% CI: 0.951–2.44, *p* = 0.08), while for PFI and DFI, higher scores were generally linked to reduced risk, reaching statistical significance for DFI (HR = 0.24, 95% CI: 0.06–0.98, *p* = 0.048). In ACRG cohort, higher FRG SignatureScore was strongly protective across all survival endpoints, including OS (HR = 0.47, 95% CI: 0.31–0.71, *p* = 0.0004), first progression (FP: HR = 0.52, 95% CI: 0.35–0.78, *p* = 0.001), and post-progression survival (PPS: HR = 0.45, 95% CI: 0.30–0.68, *p* = 0.0001). Overall, these findings indicate that the FRG SignatureScore provides robust prognostic stratification and reflects underlying molecular subtypes, although association with conventional clinicopathological features is limited. Moreover, the optimal FRG SignatureScore cutpoint varied across survival endpoints (OS, PFI/FP, and DFI), reflecting differences in event definitions, censoring patterns, and underlying biology. Despite these variations, the direction and magnitude of hazard ratios were largely consistent, supporting the strength of the FRG SignatureScore as a prognostic marker.

**Fig. 9:**
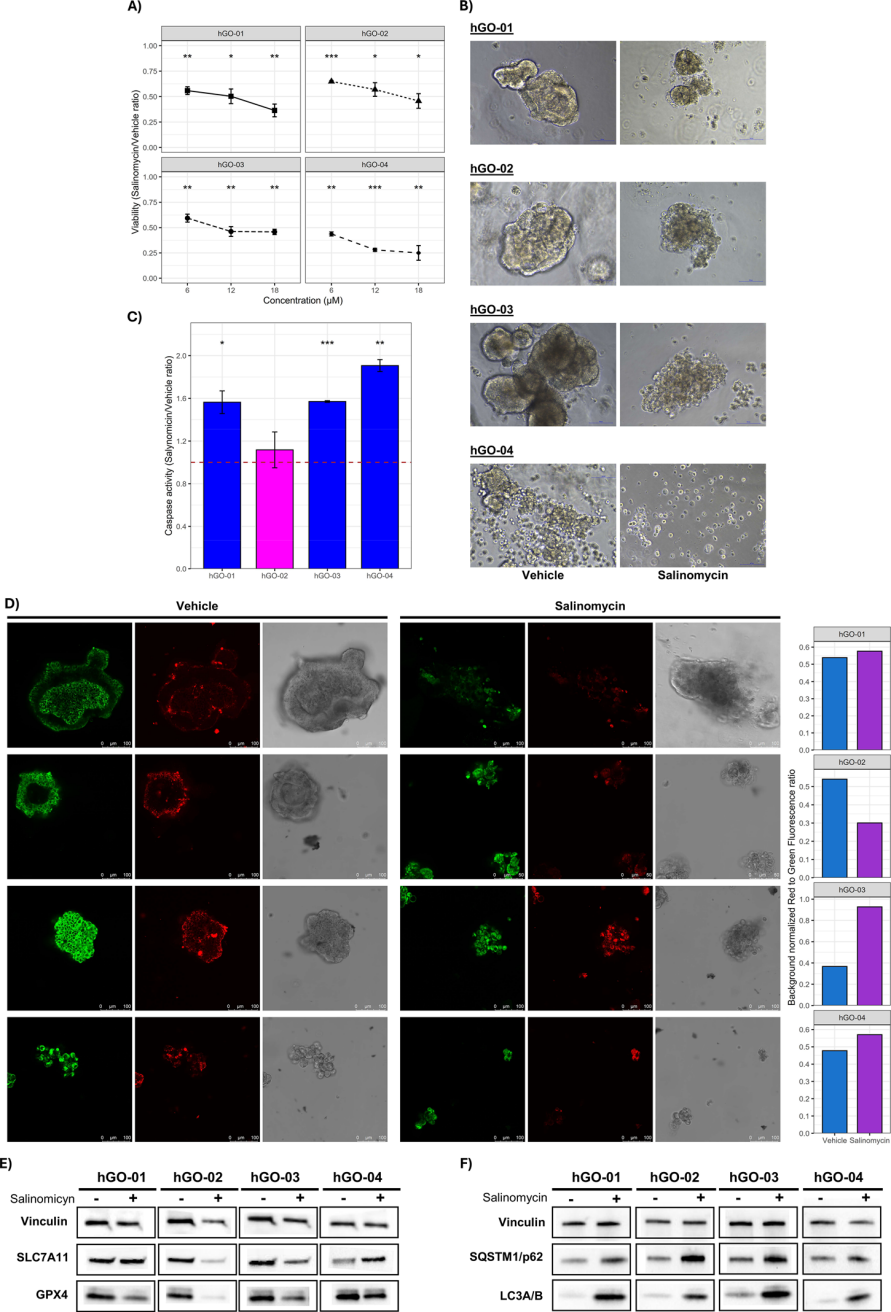
Effectiveness of Salinomycin on human Gastric Organoids (hGOs). **A** Three different concentrations of Sal were used (6, 12, 18 µM) to evaluate its effectiveness on cancerous hGOs. After 48 h of treatment significant reduction of cell viability was recorded as compared with vehicle. Three independent experiments were performed. **B** Alteration of hGOs morphology was observed after 48 h of treatment (magnification 20×) with Sal 6 µM, concentration used for all further experiments. Three independent experiments were performed. **C** Activation of apoptotic RCD was assessed by measuring caspase 3/7 activity. The plot shows the ratios between luminescence recorded in Sal *vs* vehicle treatment. Only hGO-02 did not show caspase activation. Three independent experiments were performed. **D** hGOs were treated with Sal and vehicle for 48 h and then stained with JC-1 fluorescent probe. The alteration of mitochondria membrane potential was evaluated by measuring the red to green fluorescence intensities ratio. A marked shift of this ratio was recorded for hGO-02. **E** A strong reduction of ferroptosis markers (SLC7A11 and GPX4) expression was evident in hGO-02. **F** Autophagy markers upregulation was found in all hGOs lines. Vinculin was used as housekeeping gene for both western blots (**E**, **F**). One-sample *t*-test was employed to estimate significance (*p < 0.05, **p < 0.01, ***p < 0.001).

Finally, to provide preliminary evidence of Sal’s clinical utility, we tested its anti-tumor activity on gastric cancer patient-derived organoids (PDOs). Sal treatment significantly impaired viability and compromised organoid architecture across all models (Fig. 9A, B), with apoptosis being induced in 3 out of 4 cases (Fig. 9C). This finding prompted us to evaluate ferroptosis involvement. Accordingly, we performed JC-1 staining to assess the alteration of MMP as well as western blot to estimate the expression levels of ferroptosis markers (SLC7A11 and GPX4). Notably, in the non-apoptotic PDO (hGO-02), we observed a significant shift of red to green fluorescence (lower ratio) (Fig. 8D) and the down-regulation of the ferroptosis markers SLC7A11 and GPX4 (Fig. 9E), reinforcing the evidence of Sal-induced dual lethality. Furthermore, the upregulation of SQSTM1/p62 and LC3A/B in all PDOs confirmed autophagy as a common RCD mechanism (Fig. 9F).

## Discussion

A growing body of evidence highlights the importance of RCD mechanisms, such as apoptosis and ferroptosis, in the pathogenesis and response to treatment in various cancers [20], including GC [21].

As previously argued, Sal can induce multiple forms of cell death, including apoptosis, autophagy, and ferroptosis. This positions Sal as a versatile anticancer agent with high selectivity, particularly effective in targeting and eliminating CSCs. Several studies emphasized its role as a potent ferroptosis inducer through mechanisms that increase reactive oxygen species (ROS), and promote lipid peroxidation while suppressing antioxidant defenses such as SLC7A11 and GPX4 [22, 23]. In line with this evidence, we demonstrated that Sal elicits mechanistically different RCD in distinct GC cell lines. KATO-III and AGS cells undergo ferroptosis, while NCI-N87 and SNU1 cells die by apoptosis. These results are further complicated by the fact that all cell lines exhibited autophagy, likely as early event, in response to Sal. We dissected the molecular mechanisms underlying this differential response, exploring the intricate relationships between the RCD markers listed: mTOR, SQSTM1/p62, CASP-3, PARP, GPX4, SLC7A11, BAX, LC3A/B, and survivin. Here, the central question was to identify what intrinsic cellular differences address each cell line toward a specific cell death mechanism after Sal treatment. Since cell fate is dictated by cell’s unique molecular characteristics, our study was aimed to outline a gene signature able to distinguish ferroptosis- from apoptosis-prone GC cell lines to Sal.

The fact that Sal selectively induced ferroptosis in AGS and KATO-III cells is intriguing. Ferroptosis is triggered by the accumulation of toxic lipid ROS, driving cell towards death [24–26]. Our results show a significant increase in ROS production and down-regulation of GPX4 and SLC7A11, two key markers of ferroptosis. We also found reduced expression of survivin [27, 28]. These findings indicate that Sal’s anticancer effects are mediated by targeting this crucial antioxidant axis as described in other cancer types [29, 30].

In contrast, SNU1 and NCI-N87 cells respond to Sal by undergoing apoptosis. Several studies reported that Sal treatment increases the ratio of BAX to BCL-2 [31–34], a critical step in initiating the intrinsic apoptotic pathway. Accordingly, we found increased levels of BAX, which tilts the balance towards apoptosis. Moreover, we confirmed apoptosis activation by the consumption of CASP-3 and PARP. Sal’s pro-apoptotic activity is further amplified by downregulating survivin, a powerful inhibitor of apoptosis [35].

Among the genes in the FRG signature, the upregulation of ATP5MC3, LPCAT3, and SAT1 in ferroptosis-prone cell lines is particularly noteworthy. ATP5MC3 knockdown was found to suppresses erastin-induced ferroptosis [36]. LPCAT3 is known to play a crucial role in the accumulation of lipid peroxides that defines ferroptosis [37]. SAT1 is a known regulator of lipid peroxidation and has been shown to induce ferroptosis by promoting lipid peroxidation [38]. The upregulation of these genes and others in our signature, including ABCC1 and SLC39A14 [39, 40], further supports a complex and multi-faceted molecular response to Sal that culminates in ferroptosis.

Conversely, in the apoptosis-prone cell lines, we observed a distinct pattern of gene expression. A significant down-regulation of several key ferroptosis-related genes was found, including ACSL4 and ALOX15. They are well-established drivers of ferroptosis, essential for the production of lipid peroxidation substrates and the catalysis of lipid peroxidation, respectively [41, 42]. Moreover, the down-regulation of TP53 is notable, suggesting that its reduced activity in the ferroptosis-prone cells push them toward this RCD. Overall, the dysregulation of multiple ferroptosis drivers strongly suggests that GC cells lines have their own pre-existing molecular predisposition to favor apoptosis over ferroptosis as a response to Sal. We also highlighted a strong activity of Sal against CSC. Among the GC-CSC markers, we focused on CD44 and CD133 that are known to be involved in ferroptosis regulation. CD44 is known to suppress ferroptosis by stabilizing SLC7A11 [43, 44]. Knockdown of this solute carrier was found to lower the proportion of CD44^+^ cells and CD133^+^ cells [45]. Accordingly, our flow cytometry analysis showed a marked reduction of CD44^+^ and CD133^+^ cell populations in response to Sal as well as reduced SLC7A11 levels.

By investigating the FRG SignatureScore across two independent gastric cancer patients’ cohorts, we found that its prognostic impact is strongly context-dependent. In the TCGA-STAD cohort, the FRG SignatureScore was associated to progression-related endpoints, indicating that it captures tumor-intrinsic features linked to recurrence and disease dynamics. In this context, by targeting the CSC population, Sal can be effective to reduce the risk of disease recurrence. In contrast, in the ACRG cohort, the FRG SignatureScore showed a robust and reproducible association with OS, FP, and PPS. The variation in optimal cutpoints across these endpoints is expected when using outcome-dependent optimization methods and suggests that the FRG SignatureScore captures a continuous biological gradient whose prognostic impact manifests differently across disease progression stages, consistent with the biology of stress-response pathways such as ferroptosis. Notably, the FRG SignatureScore was largely independent of conventional clinicopathological variables but was significantly associated with the EMT molecular subtype. Together, these findings suggest that the FRG SignatureScore reflects intrinsic cell death susceptibility states that are most evident in clinically homogeneous settings and for endpoints closely linked to tumor progression. The ability of Sal to induce both apoptosis and ferroptosis in gastric cancer PDOs further underscores its translational potential. Indeed, as a ‘New Approach Methodology’ (NAM) recently recognized by the FDA Modernization Act 2.0, PDOs provide a human-relevant platform that overcomes the species-specific limitations of traditional murine models while maintaining high regulatory rigor [46]. The main finding is that the cell’s molecular background acts as a decisive determinant of its final fate, which offers compelling evidence of Sal as a useful drug to target both heterogeneity and CSC in GC.

Overall, our ferroptosis-related gene (FRG) signature serves not only as a robust prognostic biomarker for gastric cancer stratification but also highlights a critical metabolic vulnerability in chemotherapy-resistant tumors. While traditional platinum-based regimens primarily rely on the induction of apoptosis, the clinical efficacy of these treatments is frequently compromised by the activation of anti-apoptotic signaling and acquired resistance mechanisms. The poor survival of apoptosis-prone patients suggests that a therapeutic strategy that relies solely on apoptotic triggers is insufficient. In this context, Sal could demonstrate significant clinical utility by exerting a “dual-strike” lethality by triggering both apoptosis and ferroptosis. This concurrent activation is clinically pivotal; by engaging the ferroptotic pathway, Sal will overcome the apoptotic blockades typical of resistant gastric cancer cells. Evidence from recent literature highlights Sal’s therapeutic potential in combination strategies. Daimon et al. showed that SAL targets MUC1‑C in cancer stem cells, dismantling the NF‑κB/MUC1‑C loop and reducing MYC-driven expression of GSR and LRP8, thereby compromising glutathione and selenium metabolism, essential for GPX4 activity [47]. Liu et al. introduced Sal-loaded nanoparticles for acute myeloid leukemia, synergistically achieving ferroptosis with venetoclax and improving safety [8]. Wang et al. demonstrated strong synergy between Sal and 5‑fluorouracil in colorectal cancer, marked by downregulation of SLC7A11/GPX4 and enhanced oxidative stress [30]. Similarly, Su et al. reported that Sal inhibits SREBP1 in clear-cell renal carcinoma, sensitizing tumors to sorafenib and linking ferroptosis to lipid metabolism [48].

Collectively, these findings confirm Sal’s multifaceted anticancer activity and its promise in overcoming drug resistance by inducing different RCD mechanisms.

## Materials and methods

### Cell culture

SNU1, NCI-N87, AGS, and KATO-III (RRID: CVCL_0099, CVCL_1603, CVCL_0139 and CVCL_0371) GC cells lines were acquired from ATCC (Manassas, VA, USA). KATO-III cells were cultured in Iscove’s Modified Dulbecco’s Medium (IMDM, GIBCO, Grand Island, NY, USA) with 1% of penicillin/streptomycin (GIBCO) and 20% (*v*/*v*) Fetal Bovine Serum (FBS) (GIBCO). NCI-N87 and SNU-1 cells were cultured in Roswell Park Memorial Institute (RPMI) medium (GIBCO) with 1% of penicillin/streptomycin (GIBCO), 10% (*v*/*v*) FBS (GIBCO) and 1% of L-Glutammine 100× (GIBCO). AGS cells were cultured in Dulbecco’s Modified Eagle Medium (DMEM) (GIBCO) containing 1% penicillin/streptomycin and 10% (*v*/*v*) FBS. All cell lines were incubated at 37 °C in 5% CO_2_ atmosphere and were split every 2–3 days routinely. All cell lines were mycoplasma free.

### Cell viability assay

Cells were plated at a density of 5 × 10^3^ cells/well into 96-well plates and grown overnight. Then, cells were treated with Salinomycin (cat. 13579, Cayman Chemical Company, Michigan, USA) at concentration ranging from 0.5 to 25 µM, using vehicle (DMSO, Sigma-Aldrich) at the highest equivalent concentration as control. In particular, the concentrations used were based on cell line sensitivity to Sal; SNU1 and AGS: 0.5, 2.5, 7.5 µM; NCI-N87: 0.5, 2.5, 7.5, 12.5 µM; KATO-III: 0.5, 2.5, 7.5, 12.5, 25 µM. To evaluate cell viability, an MTS assay (CellTiter96® AQueous One Solution, Promega, Madison, WI, USA) was employed according to manufactured instruction. Absorbance at 490 nm was measured after 1.5 h at 37 °C incubation and normalized to vehicle. The half-maximal inhibition concentration (IC50) value was estimated using the *drm* R package [49].

### Apoptosis assay

GC cell lines were treated with Sal for 48 h according to the estimated IC50 values and equivalent volumes of vehicle. GC cells were recovered by trypsinization, washed twice with cold PBS and resuspended in 100 µL of 1× binding buffer at a concentration of 1 × 10^6^ cells/mL; subsequently, 5 µL of FITC Annexin V and 5 µL Propidium Iodide (PI) (BD Biosciences, San Jose, CA, USA) were added and cells were incubated for 15 min at room temperature (RT) in the dark. Samples were analyzed using a dxFlex flow cytometer (Beckman Coulter). For each sample 10^4^ events were acquired and analyzed by Kaluza software (Beckman Coulter Diagnostics, Brea, CA, United States). Data were reported as treatment to vehicle fold change of frequencies.

### ROS fluorometric assay

ROS production in GC cells after Sal treatment was estimated by using the cell-permeant 2’,7’-dichlorodihydrofluorescein diacetate (H2DCFDA, ThermoFisher Scientific) as a ROS indicator. NCI-N87 cells were plated at a density of 4 × 10^5^ cells/well and KATO III, AGS and SNU-1 at density of 3 × 10^5^ cells/well in 6-well plates, cultured overnight and then treated with Sal IC50s. After 48 h cells were detached from the plate with 0.05% trypsin and a 10 µM H2DCFDA working solution was added. Samples were incubated at 37 °C for 30 min and centrifugate at 1500 rpm for 5 min. Subsequently, pellets were resuspended in 500 µL of PBS, incubated at 37 °C for 20 min and analyzed by dxFlex flow cytometer (Beckman Coulter). Data were analyzed by Kaluza software (Beckman Coulter Diagnostics, Brea, CA, United States) and reported as treatment to vehicle fold change of frequencies.

### Immunofluorescence staining

All cell lines were seeded into poly-lysine coated 8-well cell culture slides (SPL Life Sciences, Pocheon-si, SK) at a density of 5 × 10^4^ cells/well. After Sal treatment, medium was discarded, cells washed twice pre-cooled PBS for 5 min. Cells were fixed with 500 µL 4% paraformaldehyde at RT for 15 min, followed by permeabilization with 0.1% Triton X-100-PBS for 10 min and washed in PBS three times for 5 min each. Then, blocking solution (5% BSA in PBS) for 30 min was added and cells were incubated with anti-LC3A/B primary antibody (#4108, 1:200, Cell Signaling Technology (CST), Boston, MA, USA) and anti-SQSTM1/p62 (#5114, rabbit antibody 1:1000, CST) overnight at 4 °C in the dark. Thereafter, cells were washed three times with PBS followed by incubation with AlexaFluor488-conjugated goat anti-rabbit-IgG and AlexaFluor647-conjugated goat anti-mouse-IgG secondary antibodies in the dark at RT for 1 h. After PBS washing, cell nuclei were counterstained with Hoechst33342 dye (ProLong Glass Antifade Mountant with NucBlue, ThermoFisher Scientific) and images were captured using the Leica TCS SP8 laser confocal microscope (Leica Microsystems, Wetzlar, DE).

### Western blot analysis for apoptosis, autophagy and ferroptosis

Total proteins were extracted with RIPA buffer supplemented with 1× Protease&Phosphatase Inhibitor Cocktail (ThermoFisher Scientific). Protein Assay Dye Reagent Concentrate (Bio-Rad Laboratories, Hercules, California, USA) was used to determine proteins concentration. Protein lysates (30 µg) were resolved by SDS-PAGE, transferred to PVDF membranes (Bio-Rad), and probed with anti-human GPX4 (#52455, rabbit antibody, 1:1000, CST), SLC7A11 (MA5-35360, rabbit antibody, 1:1000 ThermoFisher Scientific), LC3A/B (#4108, rabbit antibody 1:1000, CST), SQSTM1/p62 (#5114, rabbit antibody 1:1000, CST), PARP (#9542, rabbit antibody 1:1000, CST), CASP-3 (#9662, rabbit antibody, 1:1000, CST), β-Actin (#3700, (8H10D10) mouse antibody, 1:1000, CST), α/β-Tubulin (#2148, rabbit antibody, 1:1000, CST), GAPDH (#2118, rabbit antibody, 1:1000, CST) and Vinculin (sc-73614, mouse Antibody, 1:1000, Santa Cruz Biotechnology, Dallas, Texas, USA). After thorough PBS washing, blots were incubated with HRP-conjugated anti-rabbit- or anti-mouse-IgG. Protein bands were revealed by Clarity Western ECL Substrate with ChemiDoc System and densitometry analyses were performed with ImageLab6.1 Software (Bio-Rad).

### JC-1 staining

Mitochondrial membrane potential (MMP) was assessed using the lipophilic cationic dye JC-1 (ThermoFisher Scientific). GC cell lines were plated at a density of 1 × 10^5^ cells into poly-lysine coated 170 µm Cell Imaging Dish (EppendorfAG, Hamburg, DE). After 48 h of Sal treatment, supernatant was removed, and cells were washed with 500 µL of PBS; thereafter, cells were incubated with 2 μM JC-1 for 10 min at 37 °C in the dark. After PBS, cells were analyzed using Leica TCS SP8 confocal microscope (Leica Microsystems). Fluorescence intensity was measured using three ROIs, subtracting background for both red and green fluorescence.

### Gastric cancer stemness markers expression by flow cytometry

CD44 and CD133 GC stemness markers levels were assessed. After Sal treatment, cells were harvested, centrifuged (1500 rpm for 5 min), and cell pellets was divided into two aliquots. Each aliquot was resuspended in 96 µL of Labeling Buffer (0.5% BSA + 2 mM EDTA), and 2 µL of CD133/2 anti-human-PE-REAfinity™ (Miltenyi Biotec, Bergisch Gladbach, Germany) and 2 µL of Mouse Anti-Human-CD44-FITC (BD Pharmingen, Erembodegem, BE) antibodies were added to the tubes. To set baseline fluorescence REA-Control Antibody (S), human IgG1 (Miltenyi Biotec) and Mouse IgG2b,κ (BD Pharmingen) were used as isotype controls. The samples were then vortexed and incubated in the dark at 4 °C for 10 min. The reaction was stopped using 500 µL of Labeling Buffer. The pellet obtained after centrifugation at 1500 rpm for 5 min was resuspended in 250 µL of Labeling Buffer, transferred into tubes and analyzed by dxFlex (Beckman Coulter) flow cytometer.

### Characterization of the cancer stem cell compartment: spheroid and colony-forming assays

NCI-N87 and KATO-III cells were cultured as above using Ultra-low attachment (ULA) 96-well plates. After 48 h of Sal/vehicle treatment, cells were harvested and 1000 cells/well and 500 cells/well were respectively seeded. Spheroids growth and radius were monitored over 14 days, and images were captured at days 3, 7, 10, and 14 through the Eclipse Ts2R imaging system (Nikon Europe, Amstelveen, The Netherlands) using the included software. Sphere-forming assay was also performed seeding untreated cells, which were treated at day 7 with Sal or vehicle. Spheroids growth and radius were measured before treatment and after 48 h.

To further characterize the CSC compartment, colony-forming assay was performed. As described above, treated cells were harvested and seeded in 6-well plated at a density of 12,000 (NCI-N87) and 1000 (KATO-III) cells/well. Number of plated cells was optimized considering the different colony-forming capability of each cell line. Indeed, a different efficiency was recorded, which reflects the proportion of stemness markers positive cells in the two cell lines. Efficiency, after 7 days of culture, was calculated as counted colonies to seeded cells ratio. Efficiency and average size of colonies for each seeding density were reported in Supplementary Table S4. At the same time point a very small number of colonies formed in Sal treated wells were observed. Therefore, colonies were counted after 14 days by discarding culture medium and fixing with 4% paraformaldehyde at room temperature (RT) for 10 min. After fixation, colonies were stained with crystal violet solution (0.05% in 50% methanol) for 20 min, rinsed with H_2_O and air-dried. Following the incubation period, colonies in the 6-well plates were photographed using a digital camera mounted on a fixed stand. Data analysis was performed using ImageJ software (version 1.54r, NIH, USA). Digital images were first converted to 8-bit grayscale and calibrated for spatial scale. A consistent threshold was applied to each well to distinguish colonies from the background. The “Analyze Particles” function was employed to quantify colonies, with a size exclusion filter set at >35 pixels^2^ and a circularity index of 0.1–1.0 to exclude debris and edge artifacts.

### Identification of a gene expression signature distinctive of ferroptosis-prone GC cell lines

We retrieved RNAseq data from two public repositories: Sanger Institute Cell Model Passports (https://cellmodelpassports.sanger.ac.uk/) and NCBI GEO (accession number: GSE295523), collecting only samples corresponding to the following cell lines: SNU1, NCI-N87, AGS, KATO-III, and GES-1. Gene expression data of these cell lines composed a dataset that, following pre-processing steps, was employed to perform the following comparisons: apoptosis-prone cell lines (SNU1 and NCI-N87) *vs* control (3-replicates GES-1 cell line); ferroptosis-prone cell lines (AGS and KATO-III) *vs* control. Data packaging, pre-processing, and normalizing have been carried out with *edgeR* package [50]; the trimmed mean of M-values (TMM) is the deafult normalization method chosen [51]. Differential analysis was performed using the *limma* package and the voom method to estimate the log-counts mean-variance relationship and to deliver equal or superior performance for not uniform libraries sizes [52]. Statistical significance was initially set on adjusted *p*-value < 0.05; however, to obtain a higher number of genes for further analysis, statistically significance was set at *p*-value < 0.05 and |log_2_FC| at 0.58. Accordingly, the results report a trend rather than robust statistics. The differentially expressed genes (DEGs) distinctive of each comparison were obtained intersecting the two lists to exclude common genes. All the analyses were performed by R/Bioconductor software [53, 54]. The two DEGs lists were intersected with three lists of ferroptosis-related genes, one by Hong et al. (*n* = 60) [15], and two from MSigDB gene sets (https://www.gsea-msigdb.org/gsea/msigdb), the GOBP_FERROPTOSIS (*n* = 18) and the WP_FERROPTOSIS (*n* = 64).

### Stratification of gastric cancer patients based on ferroptosis-prone gene signature

Transcriptomic and clinical data were obtained from two independent cohorts. Gene expression data (Transcripts Per Million, TPM) and corresponding clinical phenotypes of The Cancer Genome Atlas Stomach Adenocarcinoma (TCGA-STAD, *n* = 414) project were obtained from UCSC Xena Browser [55, 56]. The other cohort was obtained from the Asian Cancer Research Group (ACRG, *n* = 300) via the Gene Expression Omnibus (accession GSE62254), with clinical data obtained from Cristescu et al. [57] and outcomes data from KMplotter [58]. For the microarray-based ACRG dataset, probe IDs were mapped to gene symbols using the *hgu133plus2.db* annotation package. Probes lacking gene annotation were excluded, and expression values for genes represented by multiple probes were averaged to generate a unique expression matrix. Clinical data were curated to classify Lauren’s histological subtypes, pathologic stages (aggregated into Stage I, II, III, and IV), and outcomes. Expression values of the 20 genes in the signature were standardized to z-scores, and a specific signature score was calculated for each patient by subtracting the mean z-score of the down-regulated genes from the mean z-score of the up-regulated ones. To stratify patients into biologically distinct groups (“Ferroptosis-prone” *vs* “Apoptosis-prone”), an iterative optimization approach was employed. Potential cutoffs were screened across the 10th to 90th percentiles of the signature score distribution. The optimal cutoff was defined as the threshold yielding the lowest Holm-adjusted *p*-value in a t-test comparison between the resulting groups. Visualization of gene expression patterns was performed using heatmaps on ordered samples. Associations between the gene signature groups and clinical characteristics were evaluated using Fisher’s exact test, with *p*-values adjusted for multiple comparisons using the Holm method. In the TCGA-STAD cohort, variables included Lauren classification, therapy outcome, and pathologic stage. In the ACRG validation cohort, analysis extended to Lauren’s classification, pathologic stage, and molecular subtypes. Survival outcomes analyzed included Overall Survival (OS), Progression-Free Interval (PFI), and Disease-Free Interval (DFI) for the TCGA-STAD cohort. For the ACRG cohort, OS, First Progression (FP), and Post-Progression Survival (PPS) were evaluated. To assess prognostic value, the optimal cutpoint for the signature score was determined using maximally selected rank statistics (via the *surv_cutpoint* algorithm) to maximize the difference in survival probability. Kaplan-Meier curves were generated, and differences were assessed using the Log-rank test. Univariate Cox proportional hazards regression models were computed to determine Hazard Ratios (HR) and 95% confidence intervals.

### Evaluation of salinomycin activity on patient-derived organoids

Two patients-derived gastric cancer organoids (hGO-01 and hGO-02) were generated at our Institution (IRCCS CROB Centro di Riferimento Oncologico della Basilicata) from surgical tissue samples (Ethical Committee ref. n. 70.2018), following a standardized protocol [17, 59]. In addition, the hGO-03 and hGO-04 were purchased from ATCC (HCM_BROD-0116-C16 and HCM_BROD-0208-C16). The hGOs viability was assessed using a luminescence-based assay (CellTiter-Glow 3D Cell Viability Assay, Promega) on a multimode microplate reader (VICTOR Nivo, Revvity) as previously reported [17]. Three independent experiments were performed, using three technical replicates. The three salynomicin IC50s estimated for the cell line (6, 12, and 18 µM) were employed to identify the effective concentration. The Caspase-Glo® 3/7 Assay System was used to determine activation of apoptosis following manufacturer protocol. Briefly, hGOs were treated with 6 µM of Sal in 96-well plates. After 48 h an equal volume of reagent was added to each well, and luminescence recorded. In addition, hGOs were cultured and treated (Sal/vehicle) in ULA 24-well plates without Matrigel to obtain protein lysate and perform western blot to assess the levels of autophagy (SQSTM1/p62 and LC3ab) and ferroptosis markers (GPX4 and SLC7A11) as described above. Moreover, the effect of Sal on hGOs’ morphology as compared to vehicle was evaluated under an inverted microscope at 20× magnification after 48 h of treatment. Matrigel-free hGOs were also subjected to JC-1 staining as described above for cell lines.

### Statistical analyses

Data are presented as mean ± standard error (SE) of counts, normalized counts, or fold changes, as appropriate. Non-normally distributed data were log_2_-transformed prior to analysis. Comparisons between ratios or subgroups were performed using one-sample or two-sample *t*-tests, respectively. Statistical significance was defined as *p* < 0.05. All analyses were conducted in R (RStudio interface) [53, 54].

## Supplementary information

Supplementary information

raw western blots

Supplementary Figure 1

Supplementary File 1

## Data Availability

All data supporting the findings of this study are included in the paper and its Supplementary Information. Cell lines gene expression data were available at the following URLs: https://cellmodelpassports.sanger.ac.uk/ and https://www.ncbi.nlm.nih.gov/geo/ (accession number: GSE295523).
