# Supplementary material for: Salinomycin as a death switch: how gastric cancer cells choose their demise
Source: Cell Death Discov. 2026 Mar 24;12:171. doi: 10.1038/s41420-026-03058-2 (PMC13040004; doi:10.1038/s41420-026-03058-2)
Supplement: Supplementary file 1 — Supplementary information [file 41420_2026_3058_MOESM1_ESM.docx]

**Salinomycin as a Death Switch: How Gastric Cancer Cells Choose Their Demise**

**Running title: How Cells Die Under Salinomycin Treatment**

Pasqualina Laurenziello,^1‡^ Margherita Luongo,^1‡^ Francesca Lospinoso Severini,^1^ Giovanni Calice,^1^ Ottavia Bartolo,^1^ Geppino Falco, ^2,3^ Carlo Calabrese,^1^ Sabino Russi,^1*^ and Simona Laurino^1^

^1^ IRCCS CROB Centro di Riferimento Oncologico della Basilicata, Rionero in Vulture, PZ, Italy.

^2^ Department of Biology, University of Naples Federico II, Naples, Italy.

^3^ Biogem, Istituto di Biologia e Genetica Molecolare, Ariano Irpino (AV), Italy.

^‡^ Pasqualina Laurenziello and Margherita Luongo contributed equally to this work.

**Fig. S1. Salinomycin reduced viability of Gastric Cancer cell line.** Dose-response MTS-based assay were performed to assess the effect of Salinomycin as anti-cancer agent in four GC cell lines. Cell line specific IC50 values were obtained (Table S1). Three independent experiments were conducted. Data were analyzed as described in Material and Methods section.

**File S1. Lists of Differentially Expressed Genes.** DEGs from each comparison were included, along with both adjusted and nominal p-values. Apoptosis- and Ferroptosis-prone cell lines specific lists of DEGs were also provided. The intersection of these lists with the three ferroptosis-related gene sets was also included.

Table S1. Estimated Inhibition concentration 50 (IC50) for the different gastric cancer cell lines treated with Salinomycin.

| **Cell Line** | **Time (hours)** | **IC50*** | **t-value** | **p-value** |
| --- | --- | --- | --- | --- |
| SNU1 | 24 | 15.6 ± 9.4 | 1.6 | 0.1 |
|  | 48 | 5.9 ± 2.1 | 2.7 | 0.01 |
|  | 72 | 1.08 ± 0.4 | 3.0 | 0.005 |
| NCI-N87 | 24 | 44.6 ± 33.7 | 1.3 | 0.19 |
|  | 48 | 11.9 ± 4.1 | 2.9 | 0.006 |
|  | 72 | 5.6 ± 1.7 | 3.2 | 0.003 |
| AGS | 24 | 7.3 ± 1.1 | 6.9 | 1.2 x 10^-7^ |
|  | 48 | 6.1 ± 1.2 | 5.2 | 1.3 x 10^-5^ |
|  | 72 | 0.1 ± 1.3 | 0.1 | 0.9 |
| KATO-III | 24 | 194.1 ± 189.8 | 1.0 | 0.3 |
|  | 48 | 17.9 ± 6.1 | 2.9 | 0.005 |
|  | 72 | 5.3 ± 0.8 | 6.3 | - 1. x 10^-8^ |

* Values are reported as µM ± SE; significance: p-value < 0.05 estimated through a Log-logistic model.

Table S2. Comparison of vehicle normalized dead cells fold changes in the four gastric cancer cell lines treated with Salinomycin.

| **Cell Line** |  | **Salinomycin*** | **Vehicle*** | **Fold Change** | **p-value** |
| --- | --- | --- | --- | --- | --- |
| SNU1 | Apoptosis | 15.9 ± 3.3 | 5.4 ± 1.3 | 3.0 ± 0.2 | 0.009 |
|  | Necrosis | 5.1 ± 0.9 | 6.1 ± 0.4 | 0.8 ± 0.1 | 0.3 |
| NCI-N87 | Apoptosis | 39.8 ± 3.5 | 14.6 ± 2.7 | 2.8 ± 0.3 | 0.02 |
|  | Necrosis | 2.6 ± 0.5 | 1.1 ± 0.3 | 2.4 ± 0.2 | 0.02 |
| AGS | Apoptosis | 4.8 ± 0.3 | 4.0 ± 1.0 | 1.4 ± 0.4 | 0.4 |
|  | Necrosis | 0.8 ± 0.3 | 0.6 ± 0.2 | 1.4 ± 0.6 | 0.6 |
| KATO-III | Apoptosis | 11.1 ± 2.9 | 7.7 ± 2.3 | 1.2 ± 0.2 | 0.5 |
|  | Necrosis | 2.6 ± 0.8 | 1.9 ± 0.3 | 1.5 ± 0.8 | 0.6 |

Values are reported as mean ± SE; *: Counts (%); significance: p-value < 0.05.

Table S3. Changes in stemness markers expression in four gastric cancer cell lines following Salinomycin treatment.

| **Cell Line** | **Treatment** | **CD44^–^CD133^–^** | **FC/p** | ***CD44^+^** | **FC/p** | ***CD133^+^** | **FC/p** | **CD44^+^CD133^+^** | **FC/p** |
| --- | --- | --- | --- | --- | --- | --- | --- | --- | --- |
| SNU1 | Vehicle | 98.7 ± 0.2 | 1.0 ± 0.0 | 0.6 ± 0.1 | 1.9 ± 0.4 | 1.3 ± 0.2 | 2.3 ± 0.4 | 0.6 ± 0.1 | 1.9 ± 0.4 |
|  | Salinomycin | 97.1 ± 0.1 | 0.03 | 1.1 ± 0.1 | 0.1 | 2.9 ± 0.1 | 0.09 | 1.1 ± 0.1 | 0.1 |
| NCI-N87 | Vehicle | 66.6 ± 0.8 | 1.2 ± 0.0 | 6.0 ± 0.8 | 0.5 ± 0.1 | 33.3 ± 0.8 | 0.7 ± 0.0 | 5.9 ± 0.8 | 0.5 ± 0.1 |
|  | Salinomycin | 77.5 ± 0.5 | 0.005 | 3.0 ± 0.3 | 0.02 | 22.4 ± 0.6 | 0.002 | 2.9 ± 0.2 | 0.02 |
| AGS | Vehicle | 94.8 ± 2.2 | 0.8 ± 0.1 | 1.3 ± 0.6 | 3.7 ± 1.0 | 5.1 ± 2.1 | 4.7 ± 0.4 | 1.2 ± 0.5 | 3.9 ± 1.2 |
|  | Salinomycin | 76.7 ± 9.5 | 0.1 | 6.0 ± 4.1 | 0.1 | 23.1 ± 9.5 | 0.01 | 5.8 ± 3.4 | 0.1 |
| KATO-III | Vehicle | 12.9 ± 0.9 | 5.3 ± 0.2 | 40.8 ± 13.6 | 0.4 ± 0.1 | 85.9 ± 0.5 | 0.3 ± 0.0 | 39.6 ± 13.1 | 0.3 ± 0.0 |
|  | Salinomycin | 68.5 ± 3.1 | 0.002 | 16.8 ± 4.4 | 0.01 | 25.3 ± 1.6 | 0.0006 | 10.7 ± 2.6 | 0.005 |

Values are reported as mean counts (%) ± SE; *: sum of single- and double-positive populations; significance: p-value < 0.05. FC: Fold Change; p: p-value.

Table S4. Colony formation efficiency of NCI-N87 and KATO-III cell lines at different seeding densities.

| **Seeding Density** | **NCI-N87** | | |  | **KATO-III** | | |
| --- | --- | --- | --- | --- | --- | --- | --- |
|  | **N. of Colonies** | **Efficiency** | **Average size** |  | **N. of Colonies** | **Efficiency** | **Average size** |
| 250 | 11 | 4.4 | 68.0 |  | 188 | 75.2 | 93.4 |
| 500 | 65 | 13.0 | 96.5 |  | 393 | 78.6 | 78.8 |
| 1 000 | 177 | 17.7 | 87.1 |  | 796 | 79.6 | 95.6 |
| 3 000 | 657 | 21.9 | 102.8 |  | 2 626 | 87.5 | 106.7 |
| 6 000 | 1 131 | 18.8 | 91.9 |  | 2 818 | 46.9 | 139.7 |
| 12 000 | 1 976 | 16.5 | 123.8 |  | 3 954 | 32.9 | 205.2 |
