# Supplementary material for: Salinomycin as a death switch: how gastric cancer cells choose their demise
Source: Cell Death Discov. 2026 Mar 24;12:171. doi: 10.1038/s41420-026-03058-2 (PMC13040004; doi:10.1038/s41420-026-03058-2)
Supplement: Supplementary file 2 — raw western blots [file 41420_2026_3058_MOESM2_ESM.pptx]

## Slide 1
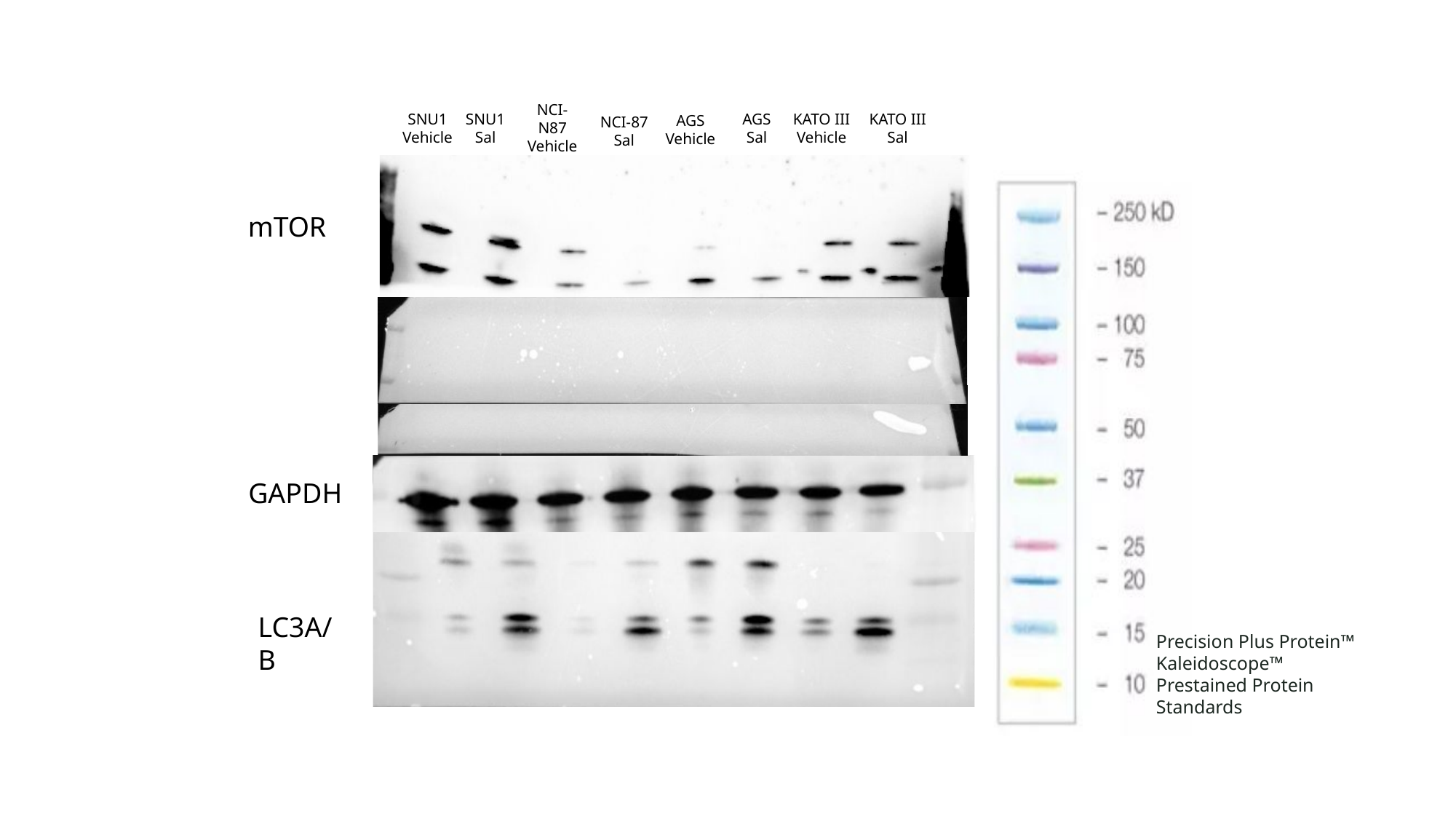

SNU1
Vehicle
NCI-N87
Vehicle
SNU1
Sal
AGS
Sal
KATO III
Vehicle
KATO III
Sal
AGS
Vehicle
NCI-87
Sal
mTOR
GAPDH
LC3A/B
Precision Plus Protein™ Kaleidoscope™ Prestained Protein Standards

## Slide 2
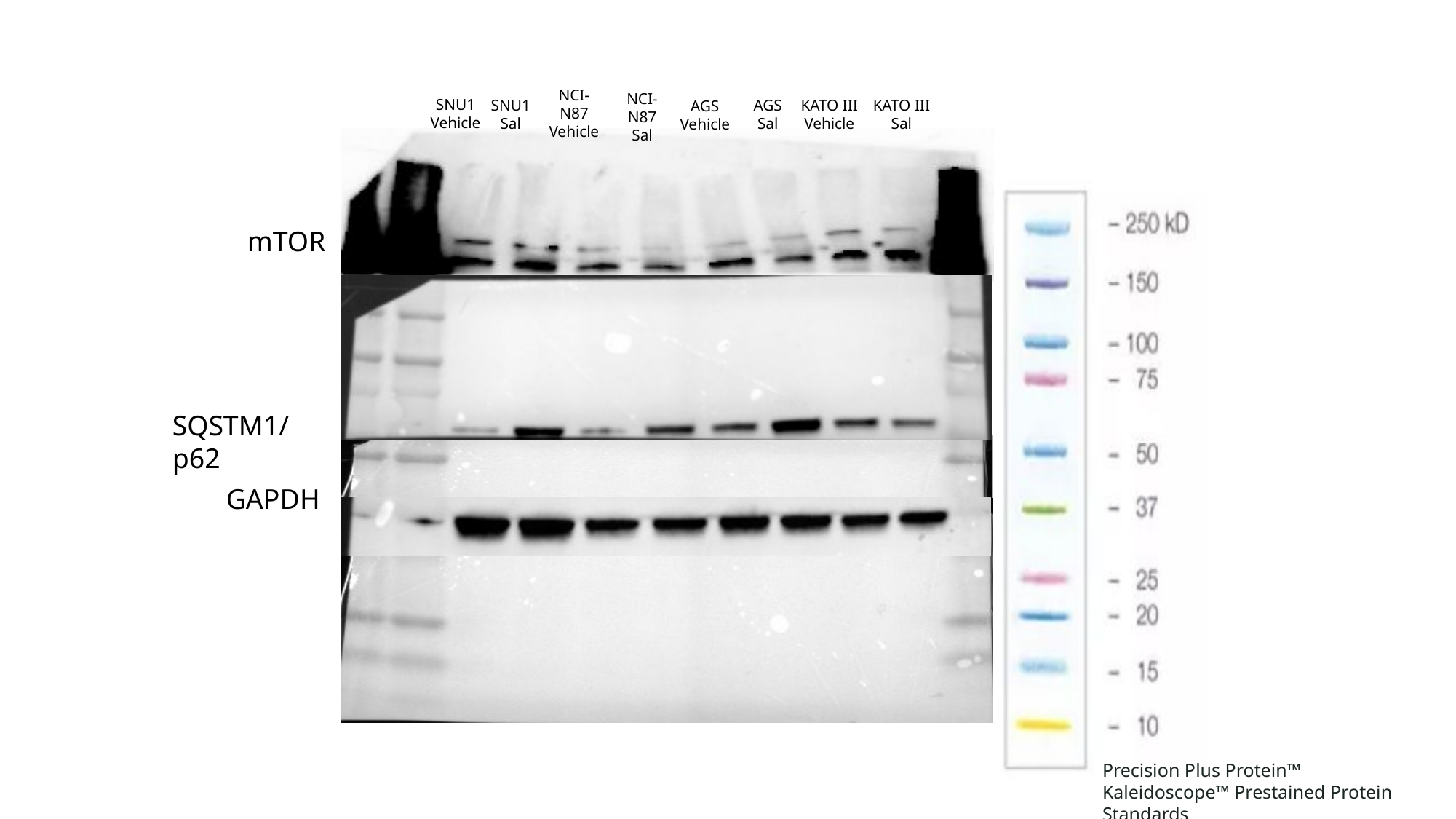

NCI-N87
Vehicle
NCI-N87
Sal
SNU1
Vehicle
SNU1
Sal
AGS
Sal
KATO III
Vehicle
KATO III
Sal
AGS
Vehicle
mTOR
SQSTM1/p62
GAPDH
Precision Plus Protein™ Kaleidoscope™ Prestained Protein Standards

## Slide 3
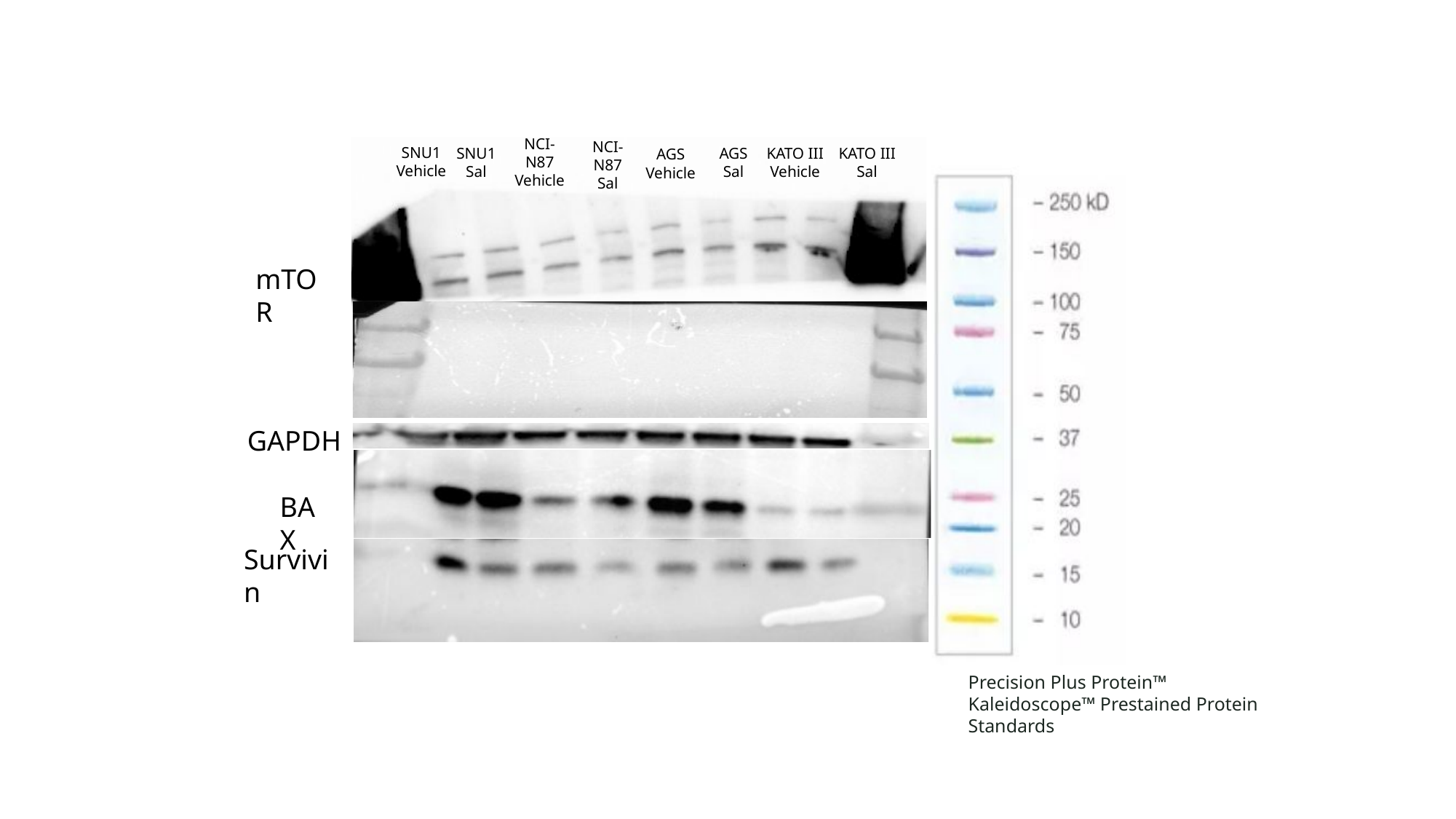

NCI-N87
Vehicle
NCI-N87
Sal
SNU1
Vehicle
SNU1
Sal
AGS
Sal
KATO III
Vehicle
KATO III
Sal
AGS
Vehicle
mTOR
GAPDH
BAX
Survivin
Precision Plus Protein™ Kaleidoscope™ Prestained Protein Standards

## Slide 4
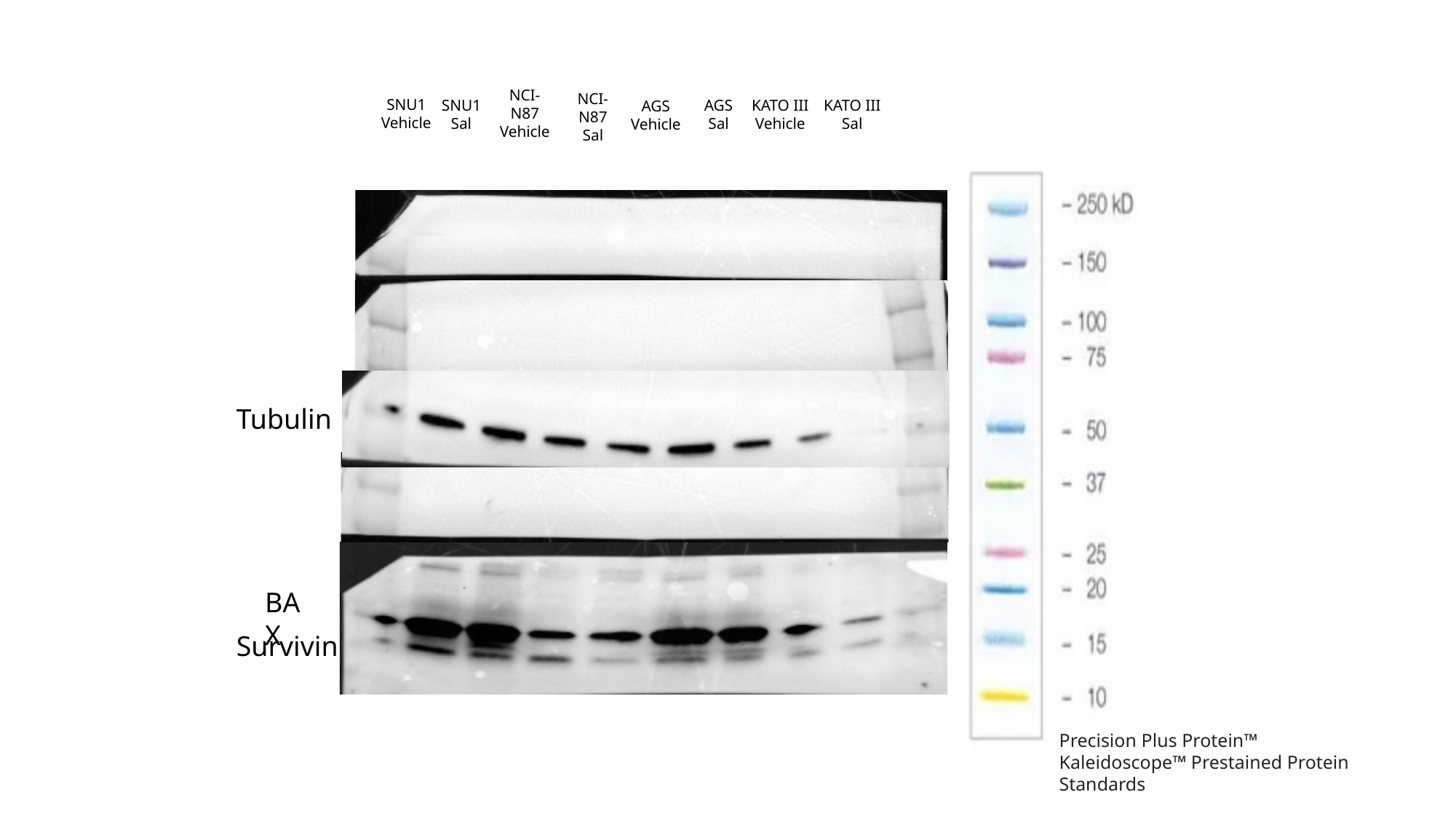

NCI-N87
Vehicle
NCI-N87
Sal
SNU1
Vehicle
SNU1
Sal
AGS
Sal
KATO III
Vehicle
KATO III
Sal
AGS
Vehicle
Tubulin
BAX
Survivin ​
Precision Plus Protein™ Kaleidoscope™ Prestained Protein Standards

## Slide 5
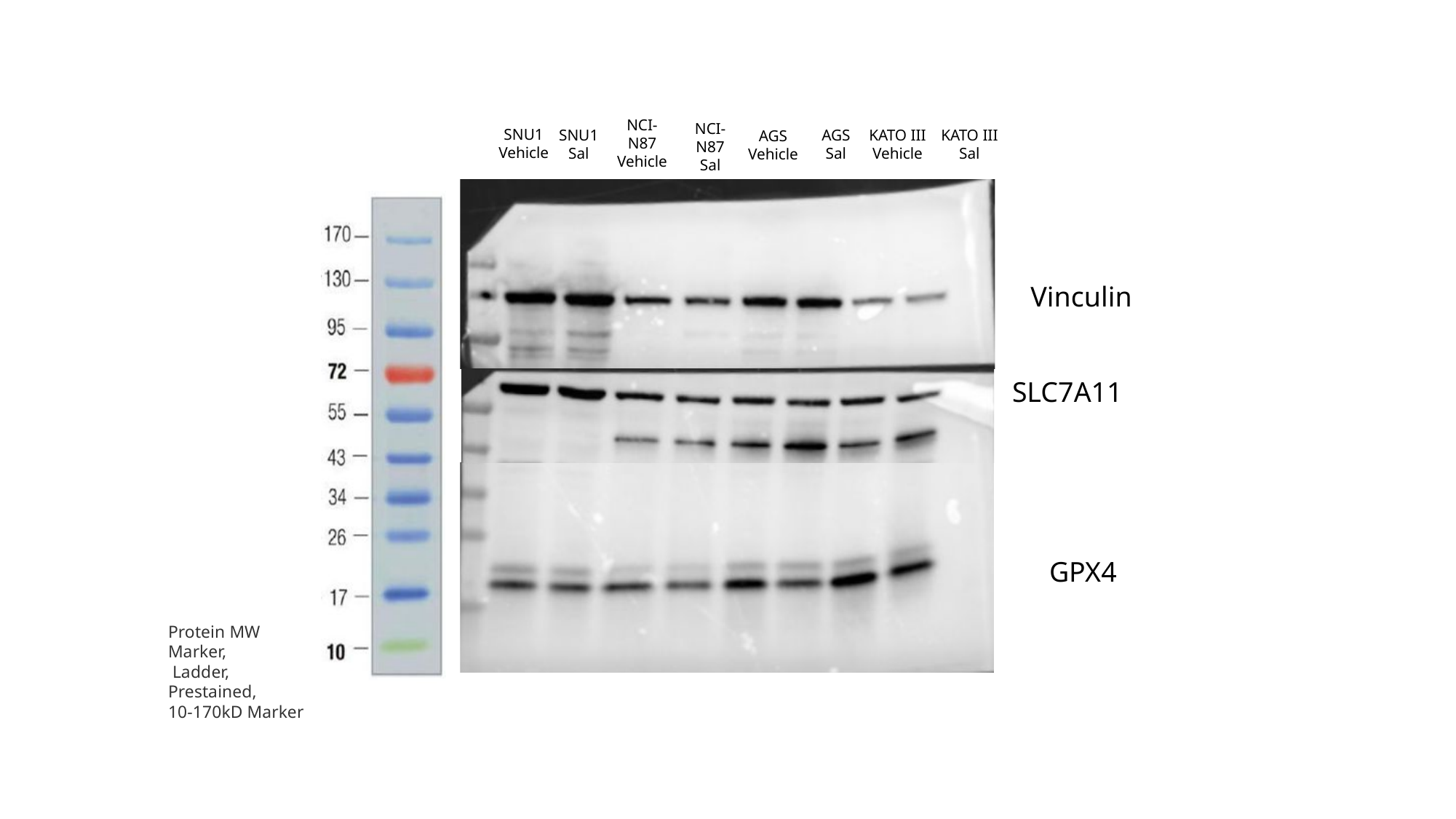

NCI-N87
Vehicle
NCI-N87
Sal
SNU1
Vehicle
SNU1
Sal
AGS
Sal
KATO III
Vehicle
KATO III
Sal
AGS
Vehicle
Vinculin
SLC7A11
GPX4
Protein MW Marker,
 Ladder, Prestained,
10-170kD Marker

## Slide 6
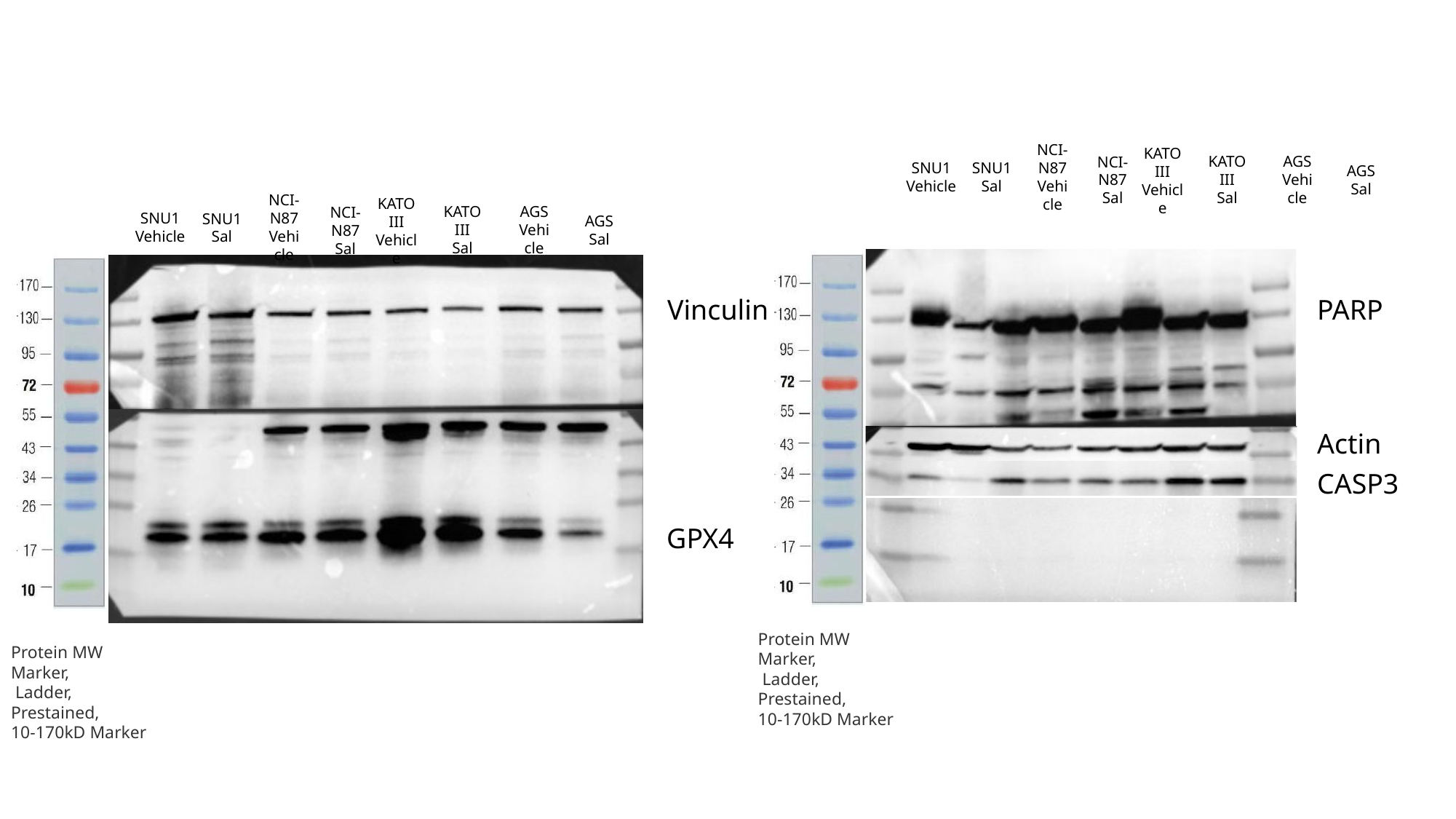

NCI-N87
Vehicle
NCI-N87
Sal
SNU1
Vehicle
SNU1
Sal
AGS
Vehicle
AGS
Sal
KATO III
Sal
KATO III
Vehicle
NCI-N87
Vehicle
NCI-N87
Sal
SNU1
Vehicle
SNU1
Sal
AGS
Vehicle
AGS
Sal
KATO III
Sal
KATO III
Vehicle
Vinculin
PARP
Actin
CASP3
GPX4
Protein MW Marker,
 Ladder, Prestained,
10-170kD Marker
Protein MW Marker,
 Ladder, Prestained,
10-170kD Marker

## Slide 7
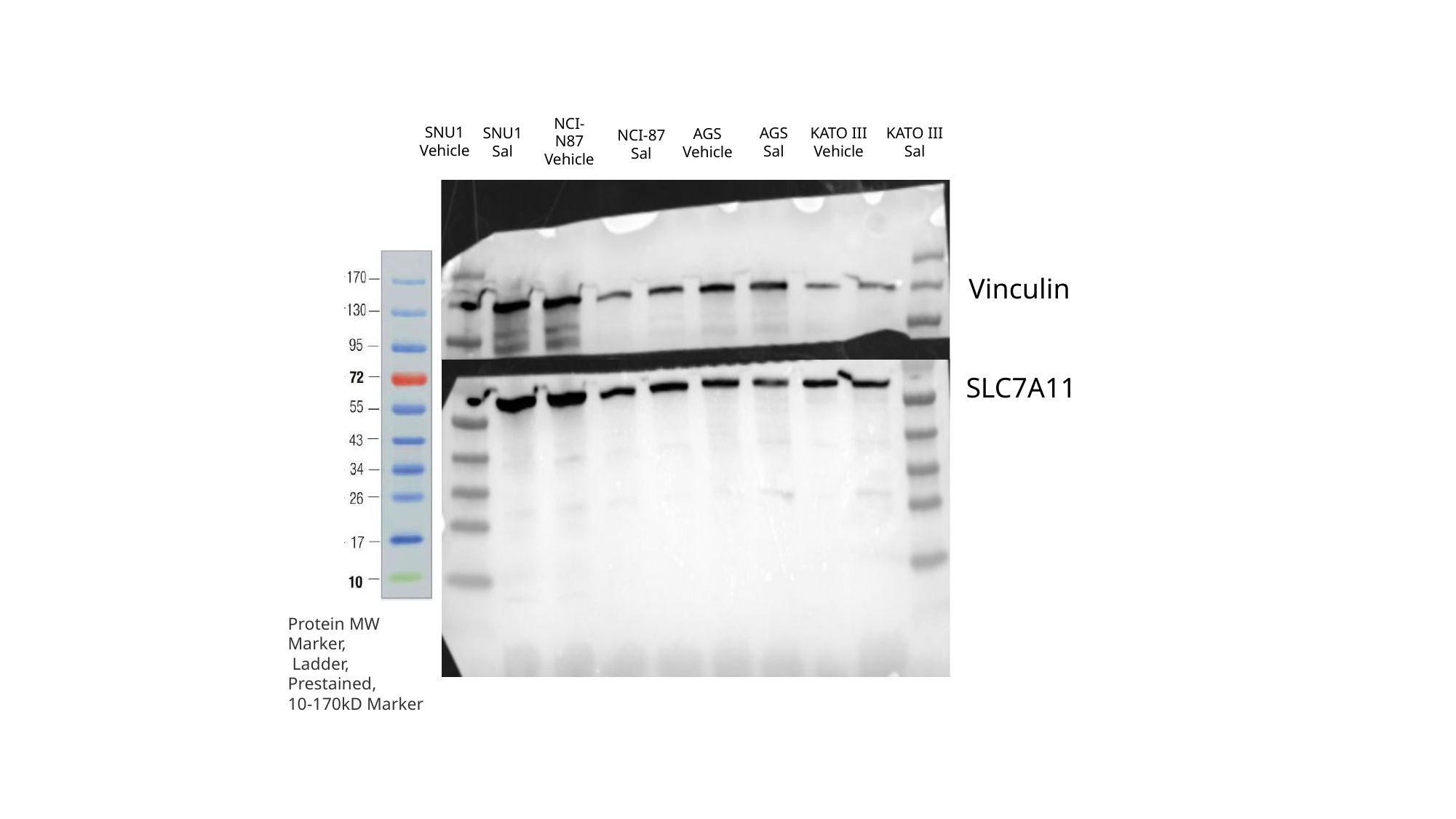

SNU1
Vehicle
NCI-N87
Vehicle
SNU1
Sal
AGS
Sal
KATO III
Vehicle
KATO III
Sal
AGS
Vehicle
NCI-87
Sal
Vinculin
SLC7A11
Protein MW Marker,
 Ladder, Prestained,
10-170kD Marker

## Slide 8
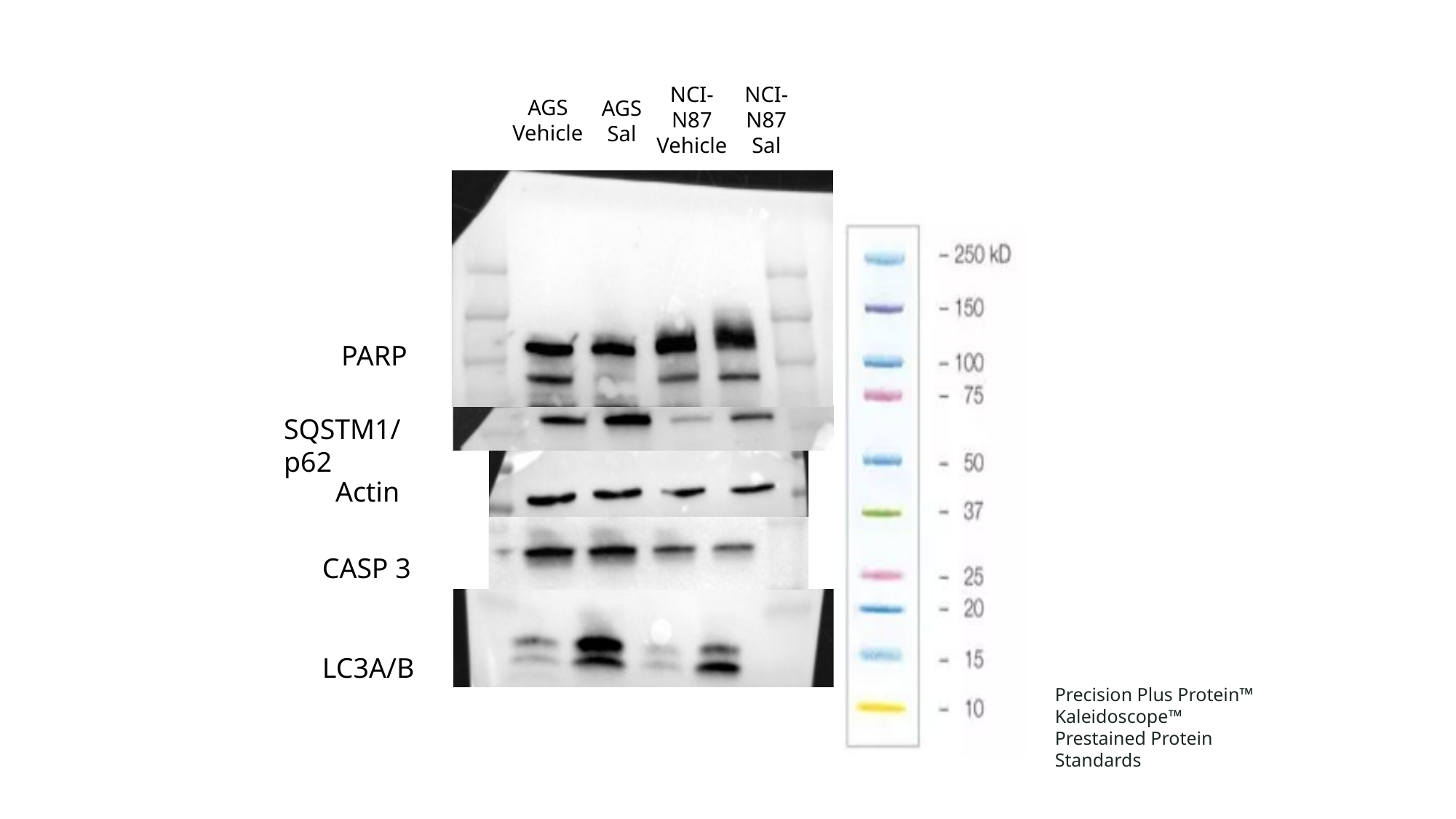

NCI-N87
Vehicle
NCI-N87
Sal
AGS
Vehicle
AGS
Sal
PARP
SQSTM1/p62
Actin
CASP 3
LC3A/B
Precision Plus Protein™ Kaleidoscope™ Prestained Protein Standards

## Slide 9
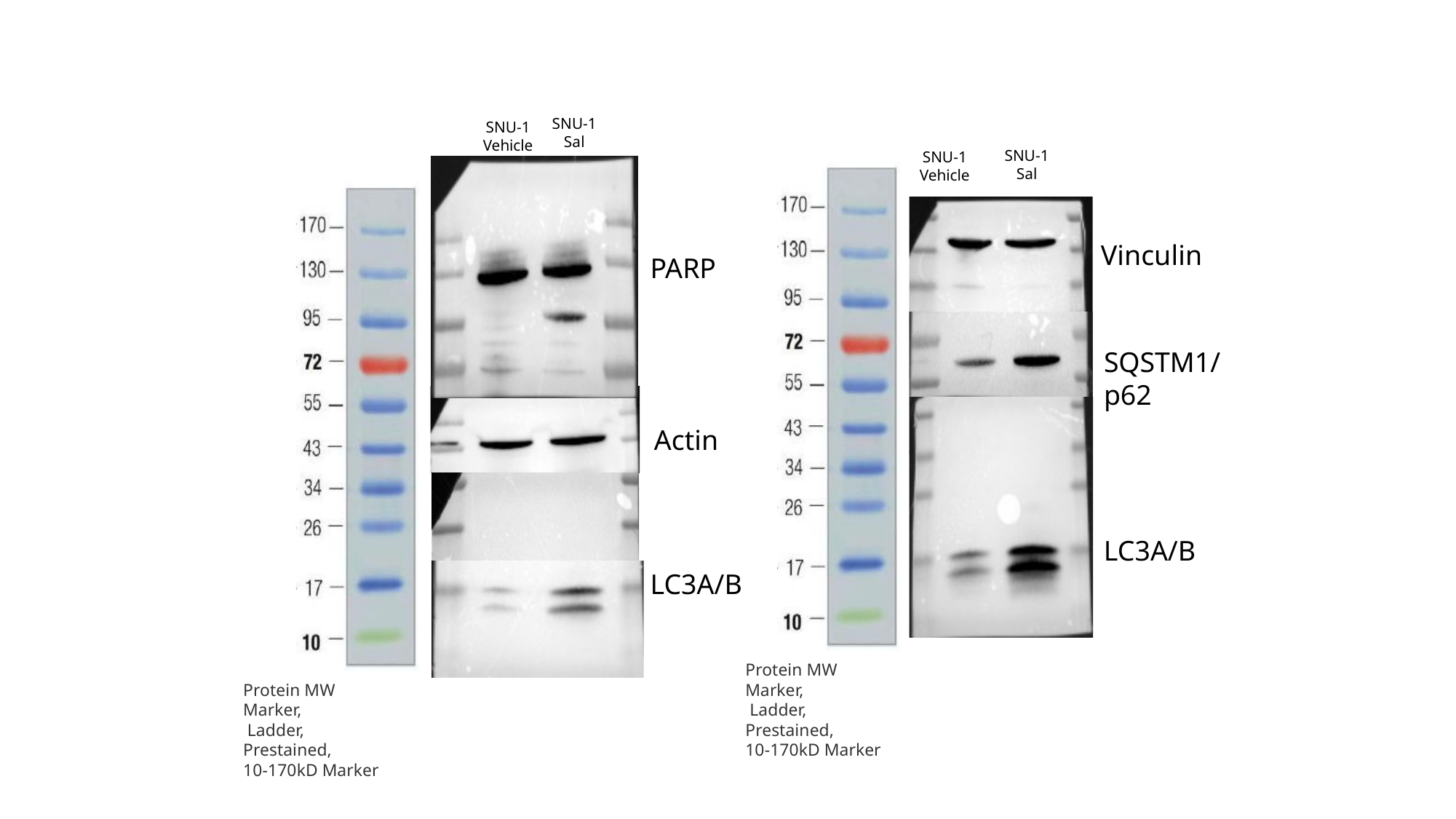

SNU-1
Sal
SNU-1
Vehicle
SNU-1
Sal
SNU-1
Vehicle
Vinculin
PARP
SQSTM1/p62
Actin
LC3A/B
LC3A/B
Protein MW Marker,
 Ladder, Prestained,
10-170kD Marker
Protein MW Marker,
 Ladder, Prestained,
10-170kD Marker

## Slide 10
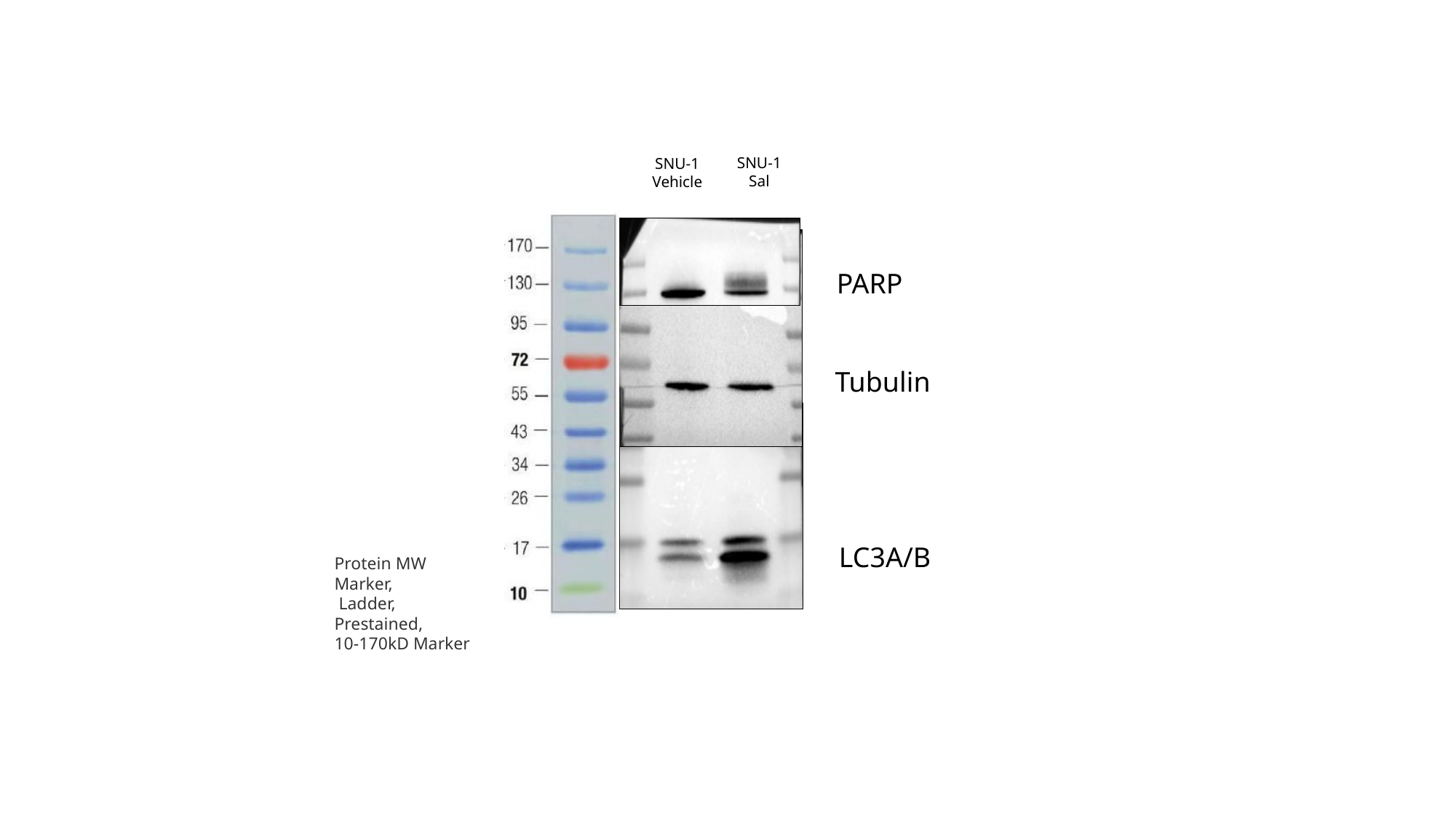

SNU-1
Sal
SNU-1
Vehicle
Protein MW Marker,
 Ladder, Prestained,
10-170kD Marker
PARP
Tubulin
LC3A/B

## Slide 11
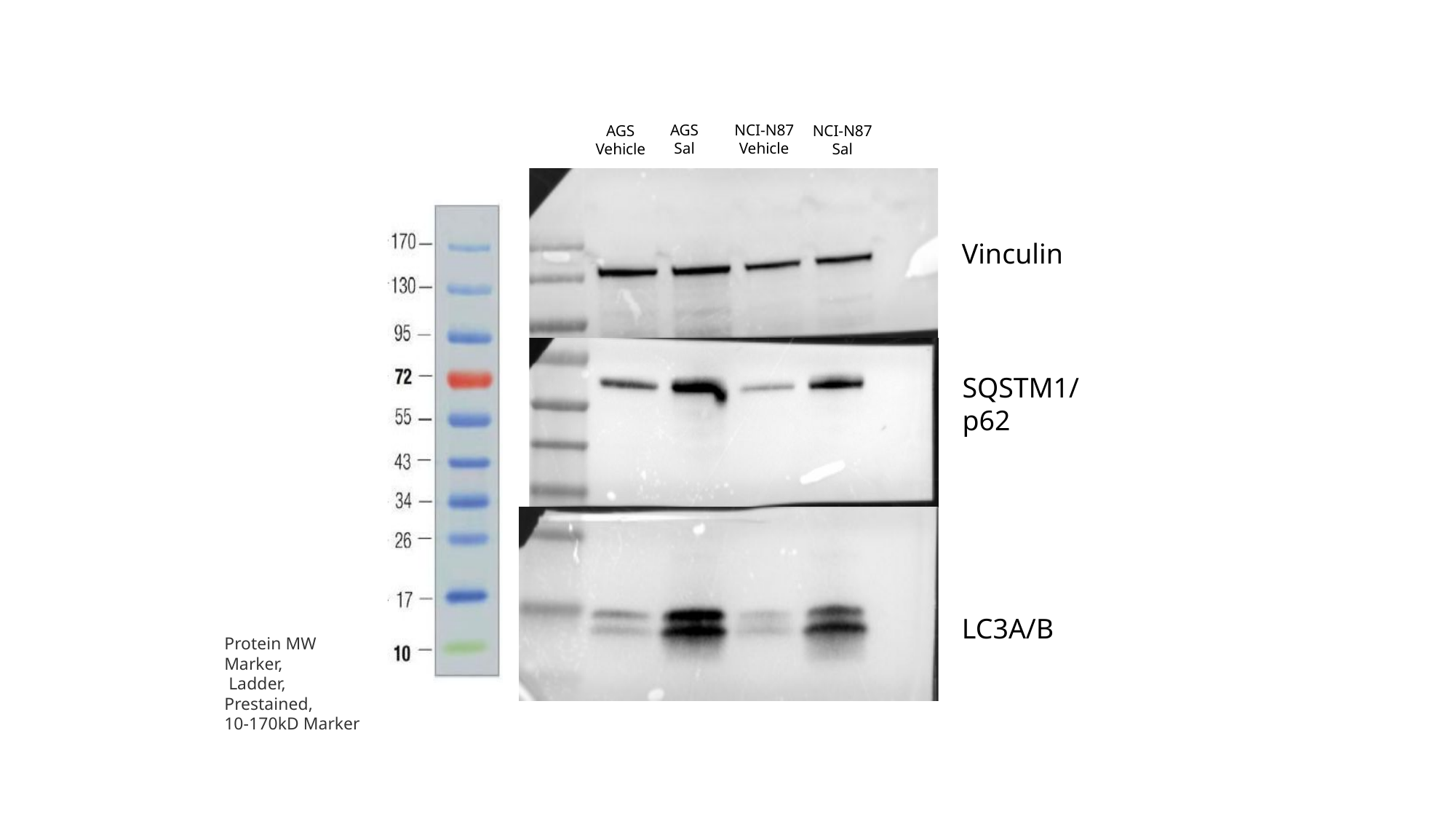

AGS
Sal
NCI-N87
Vehicle
AGS
Vehicle
NCI-N87
Sal
Protein MW Marker,
 Ladder, Prestained,
10-170kD Marker
Vinculin
SQSTM1/p62
LC3A/B

## Slide 12
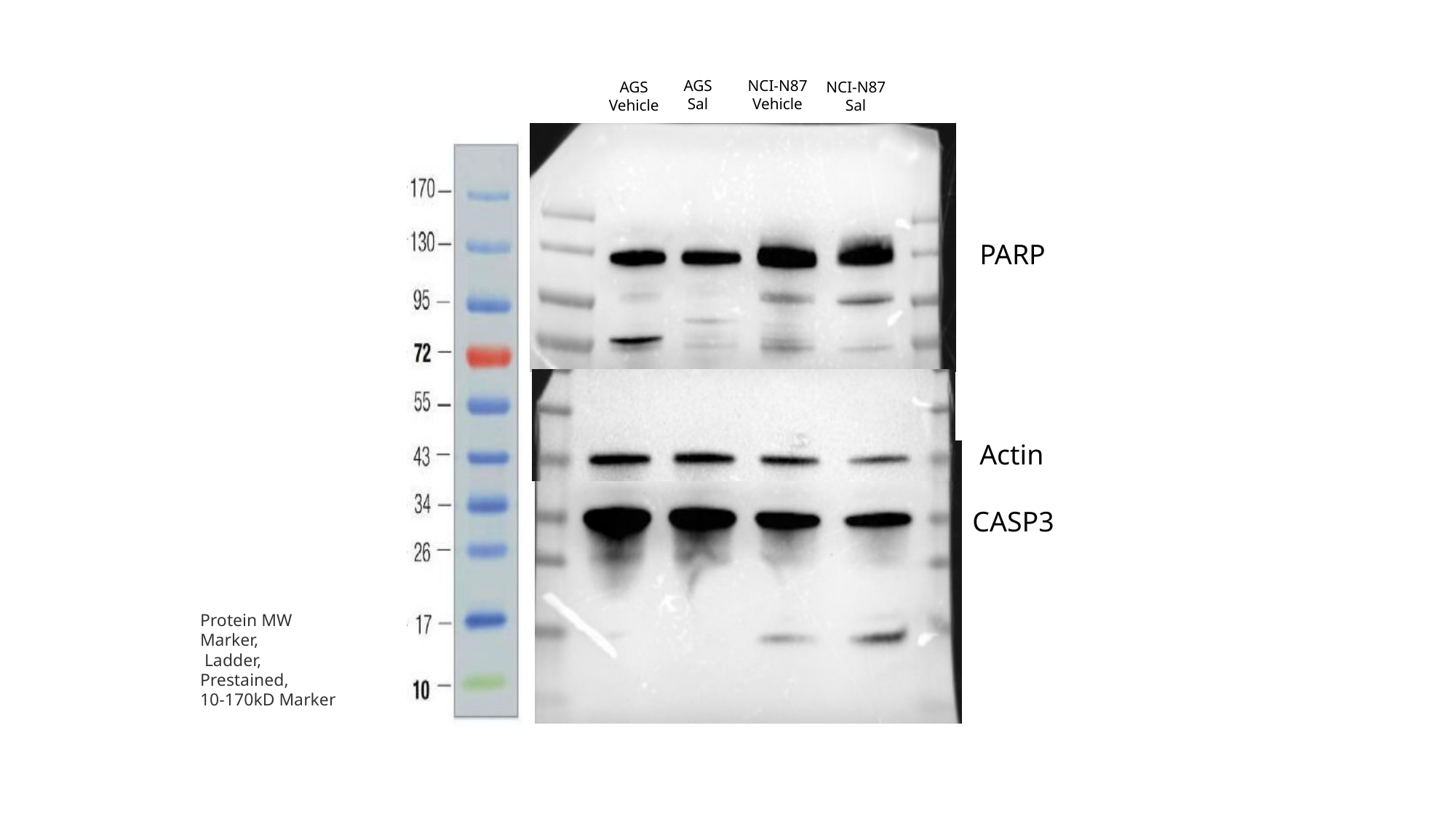

AGS
Sal
NCI-N87
Vehicle
AGS
Vehicle
NCI-N87
Sal
Protein MW Marker,
 Ladder, Prestained,
10-170kD Marker
PARP
Actin
CASP3

## Slide 13
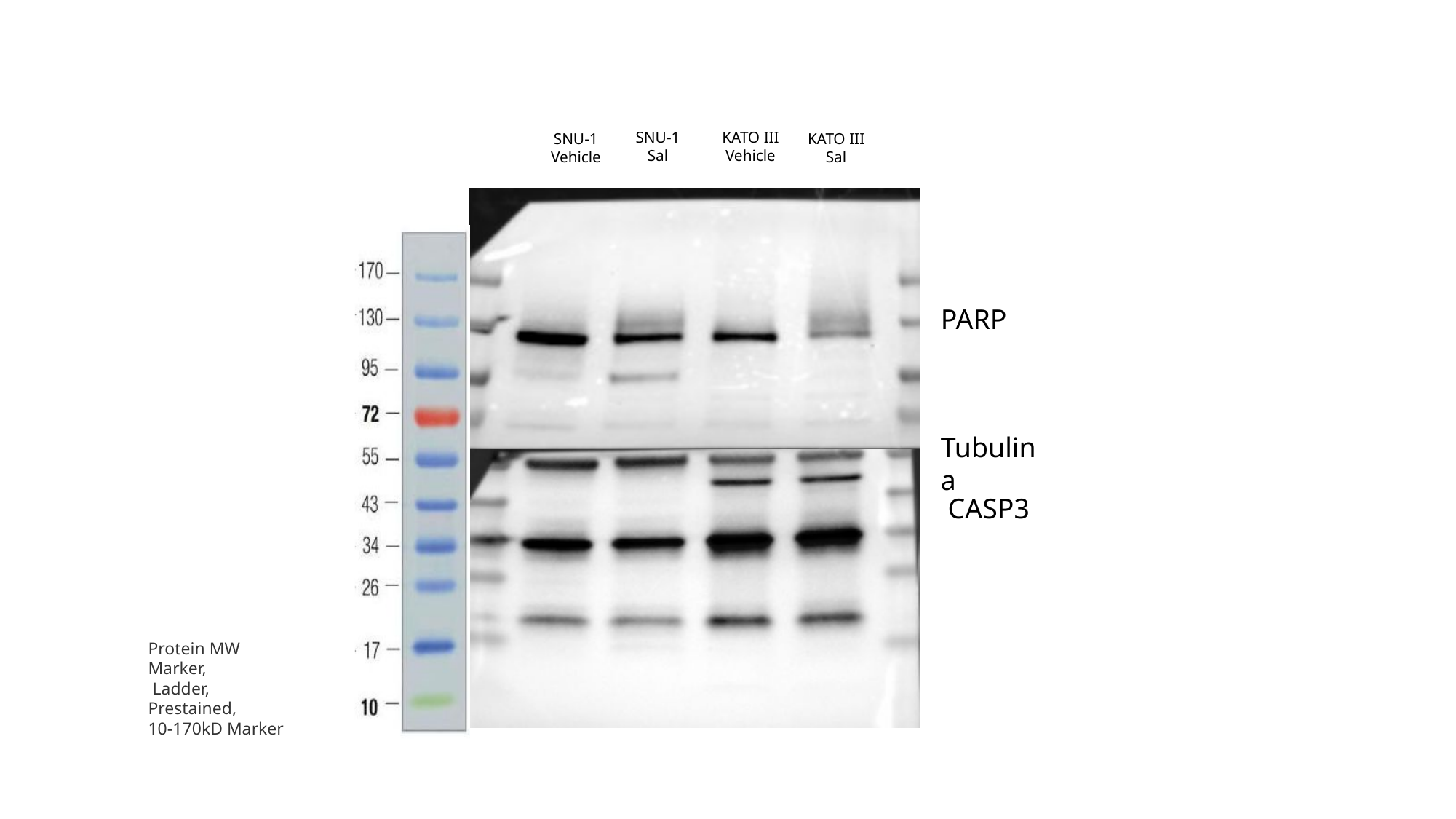

SNU-1
Sal
KATO III
Vehicle
SNU-1
Vehicle
KATO III
Sal
Protein MW Marker,
 Ladder, Prestained,
10-170kD Marker
PARP
Tubulina
CASP3

## Slide 14
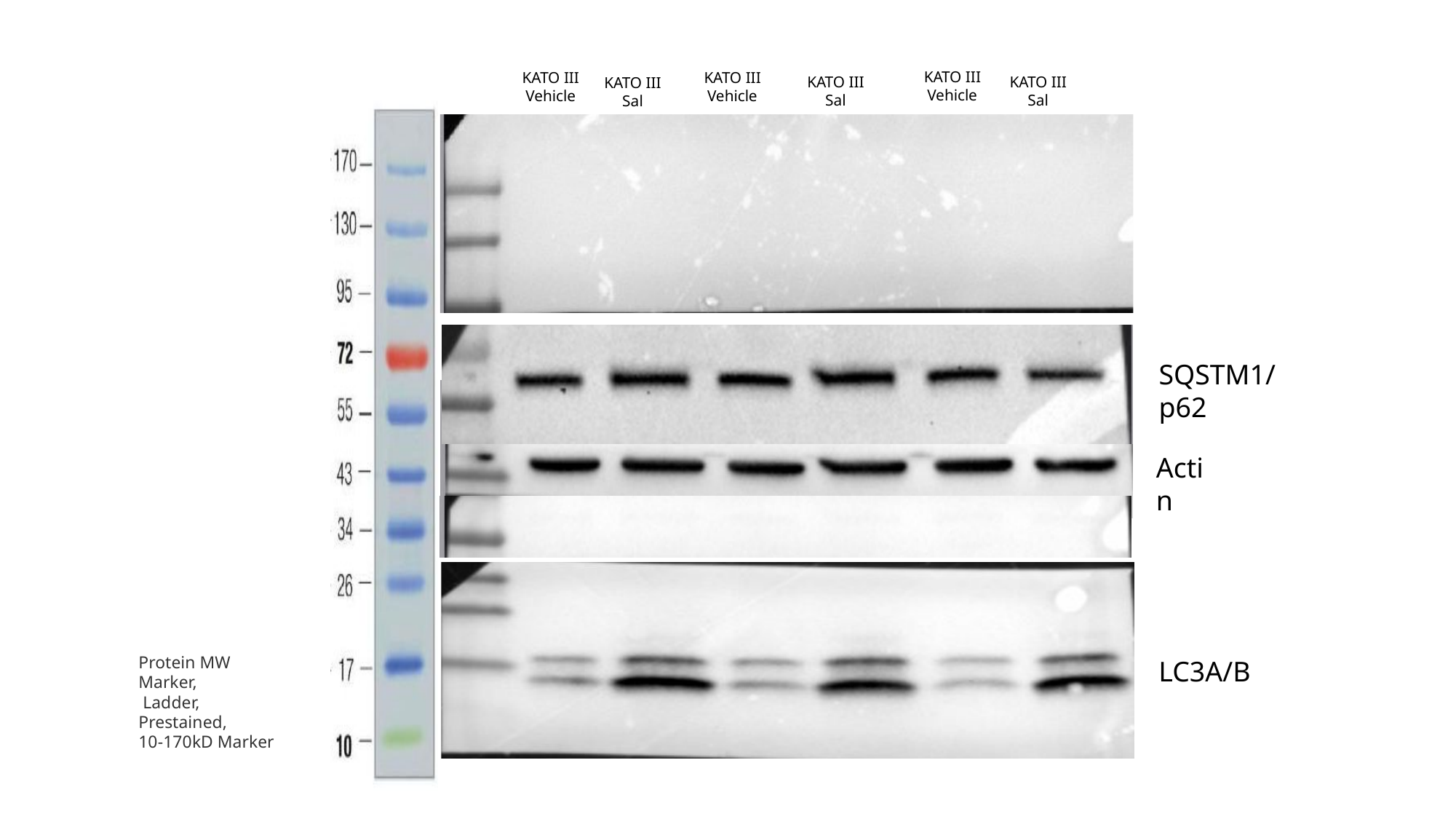

KATO III
Vehicle
KATO III
Vehicle
KATO III
Vehicle
KATO III
Sal
KATO III
Sal
KATO III
Sal
Protein MW Marker,
 Ladder, Prestained,
10-170kD Marker
SQSTM1/p62
Actin
LC3A/B

## Slide 15
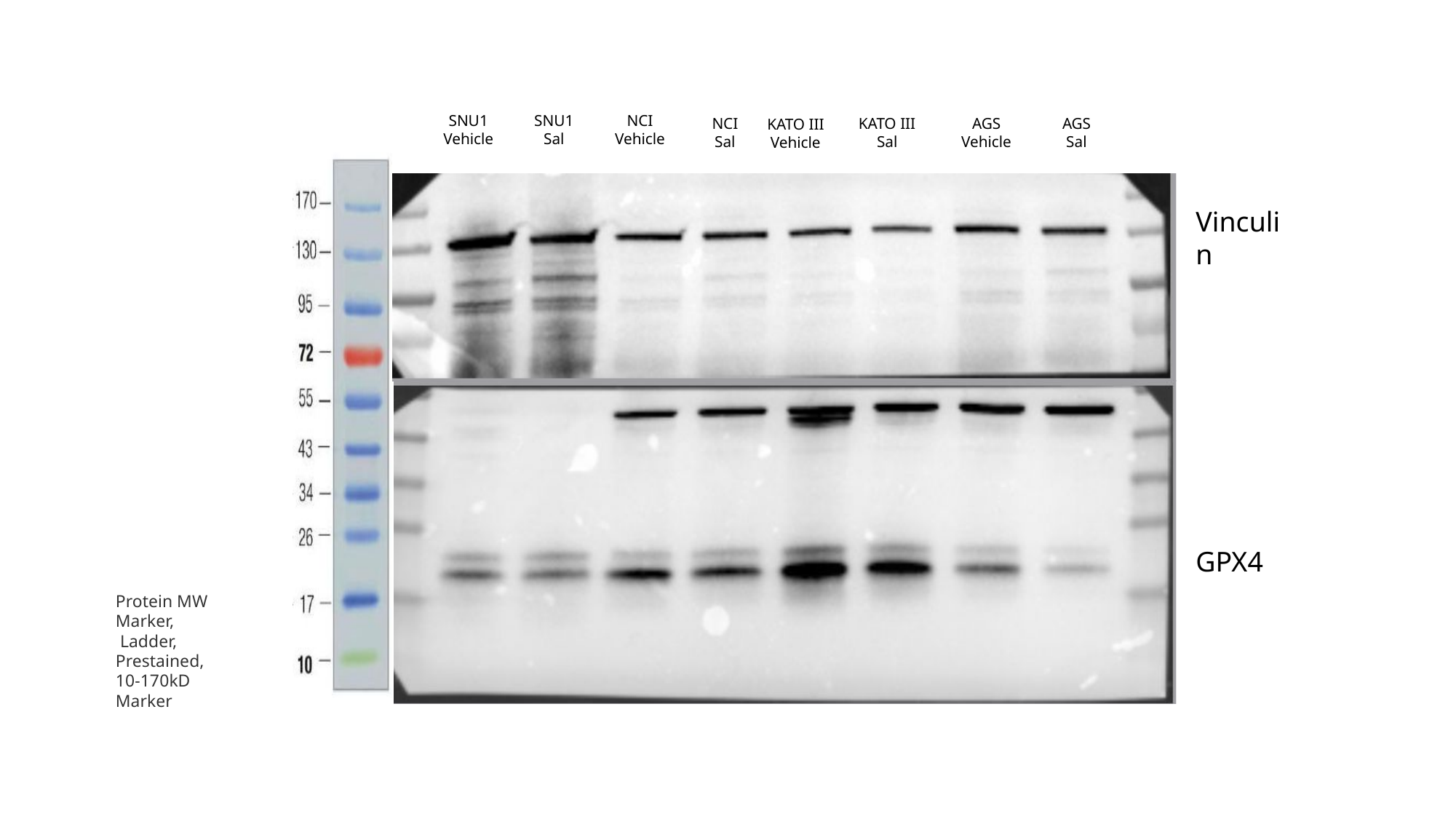

SNU1
Vehicle
NCI
Vehicle
SNU1
Sal
AGS
Vehicle
AGS
Sal
KATO III
Sal
NCI
Sal
KATO III
Vehicle
Protein MW Marker,
 Ladder, Prestained,
10-170kD Marker
Vinculin
GPX4

## Slide 16
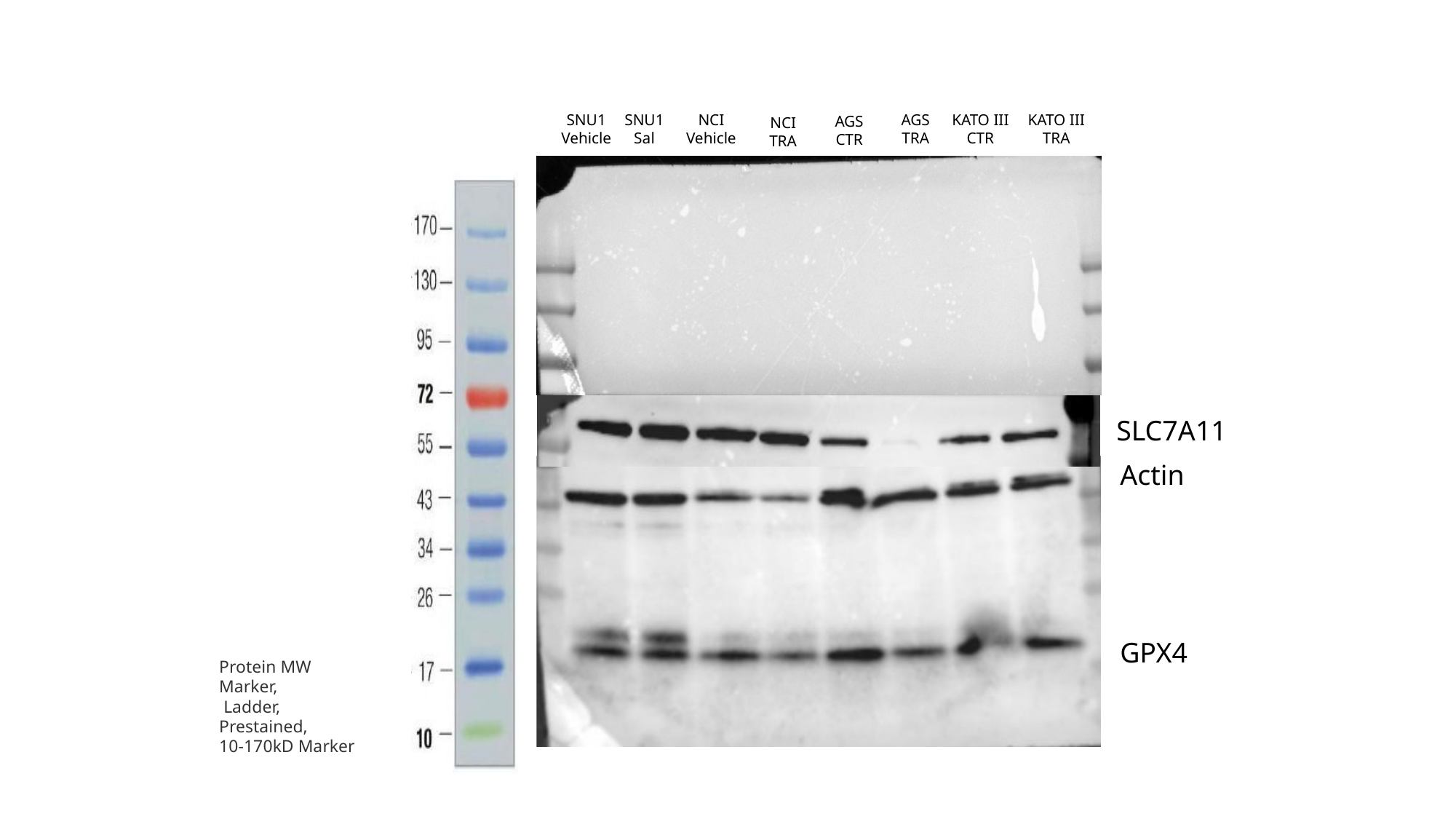

SNU1
Vehicle
NCI
Vehicle
SNU1
Sal
AGS
TRA
KATO III
CTR
KATO III
TRA
AGS
CTR
NCI
TRA
Protein MW Marker,
 Ladder, Prestained,
10-170kD Marker
SLC7A11
Actin
GPX4

## Slide 17
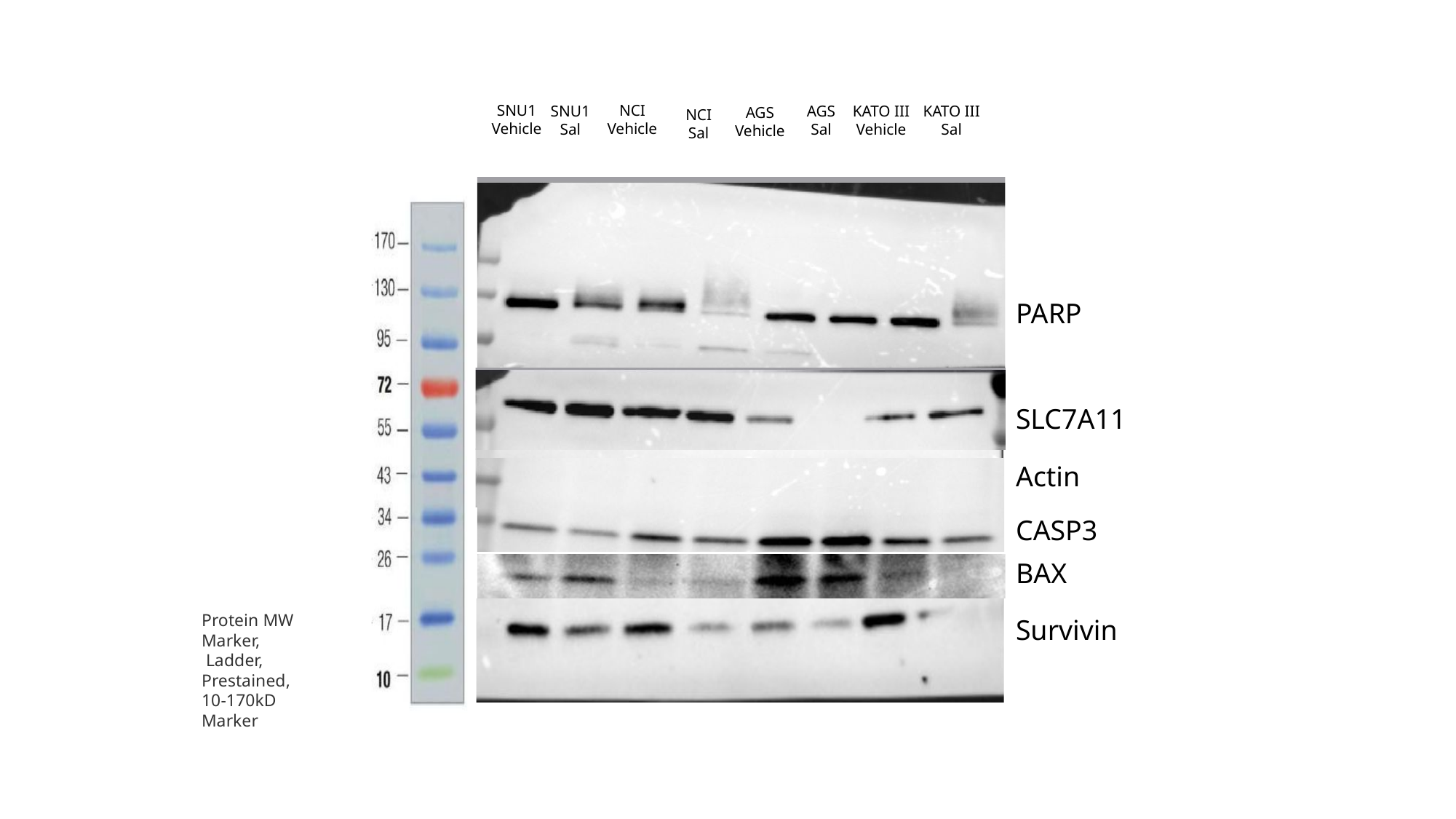

SNU1
Vehicle
NCI
Vehicle
SNU1
Sal
AGS
Sal
KATO III
Vehicle
KATO III
Sal
AGS
Vehicle
NCI
Sal
Protein MW Marker,
 Ladder, Prestained,
10-170kD Marker
PARP
SLC7A11
Actin
CASP3
BAX
Survivin

## Slide 18
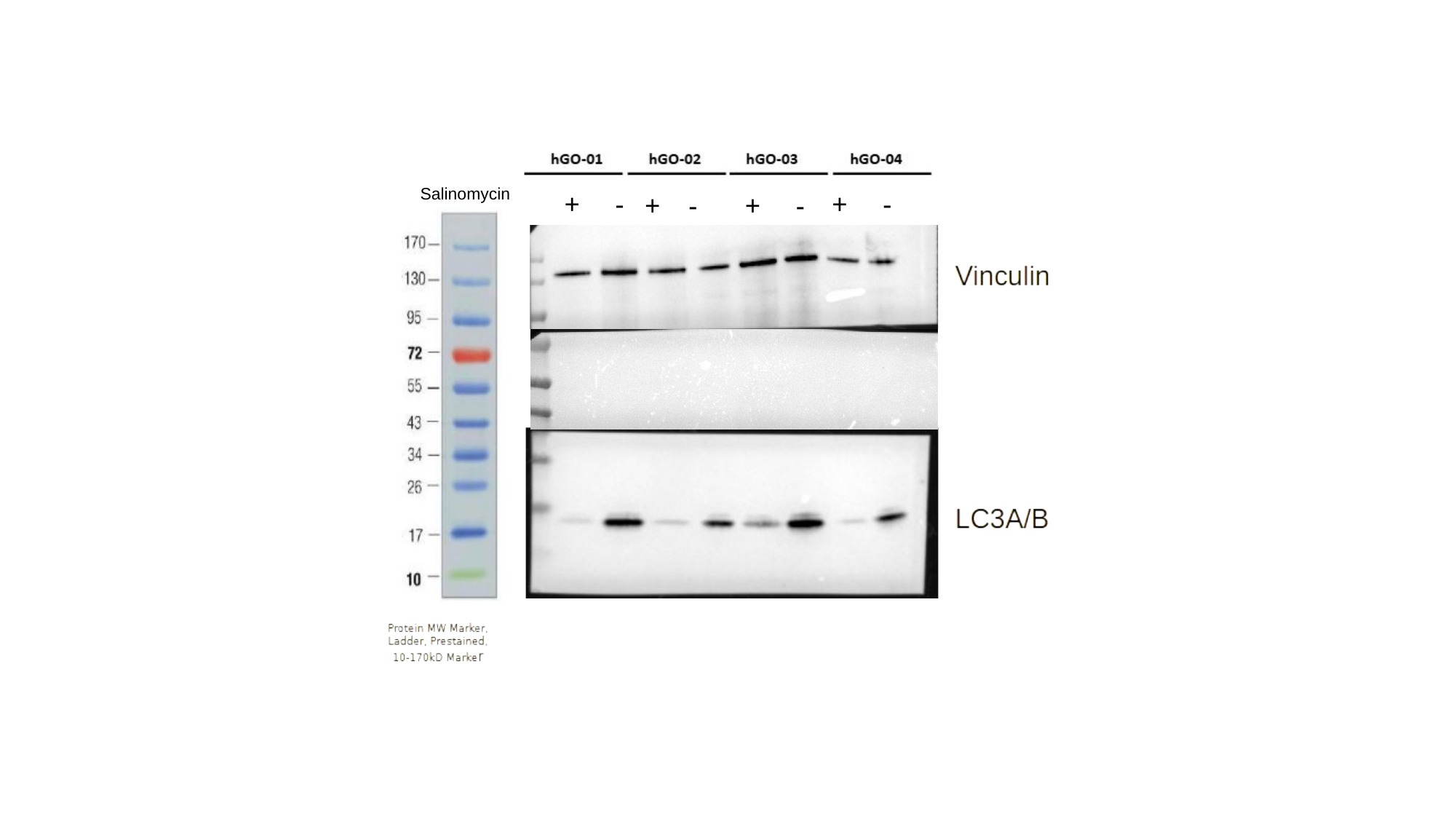

Salinomycin
+ -
+ -
+ -
+   -

## Slide 19
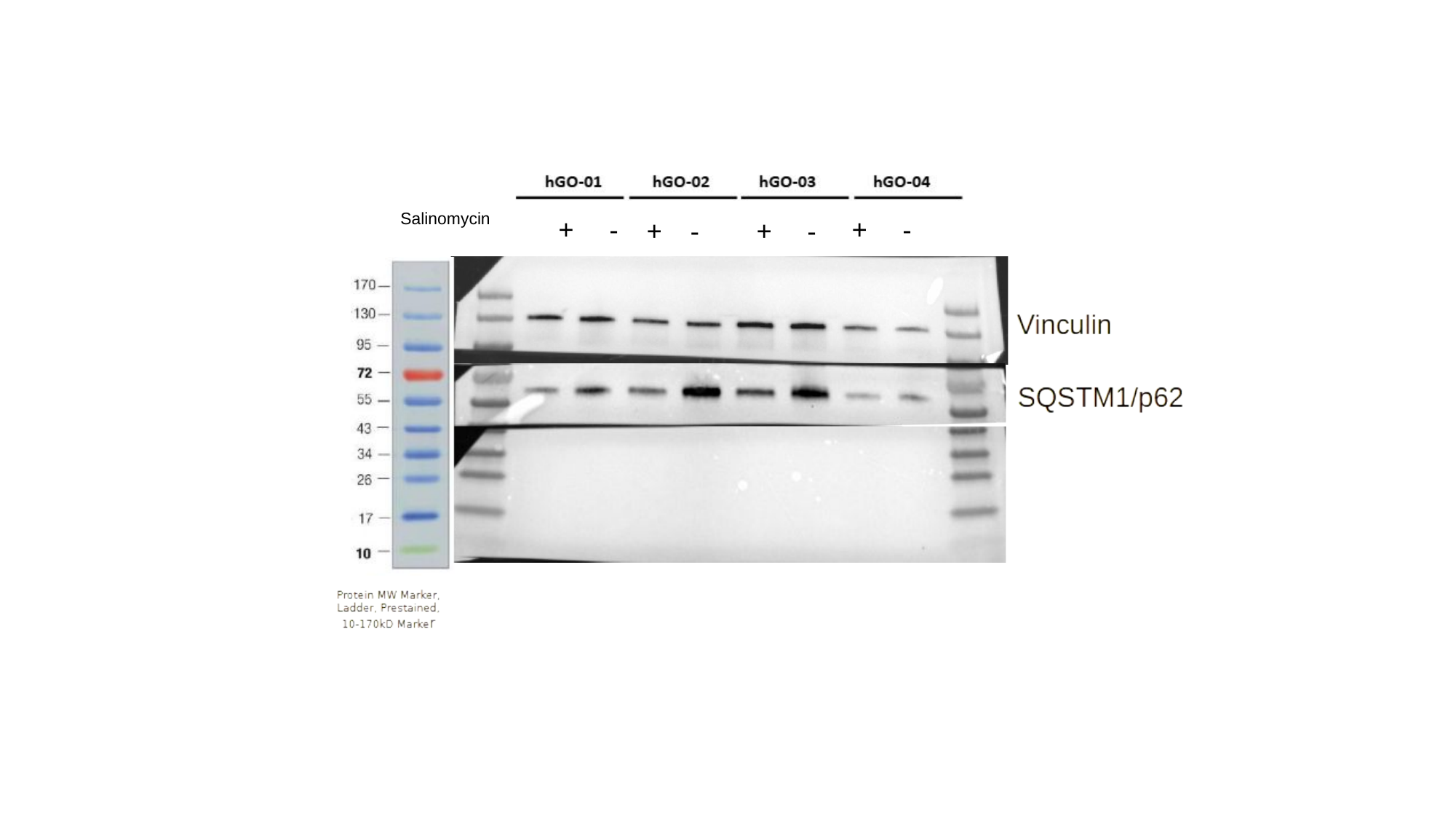

Salinomycin
+ -
+ -
+ -
+   -

## Slide 20
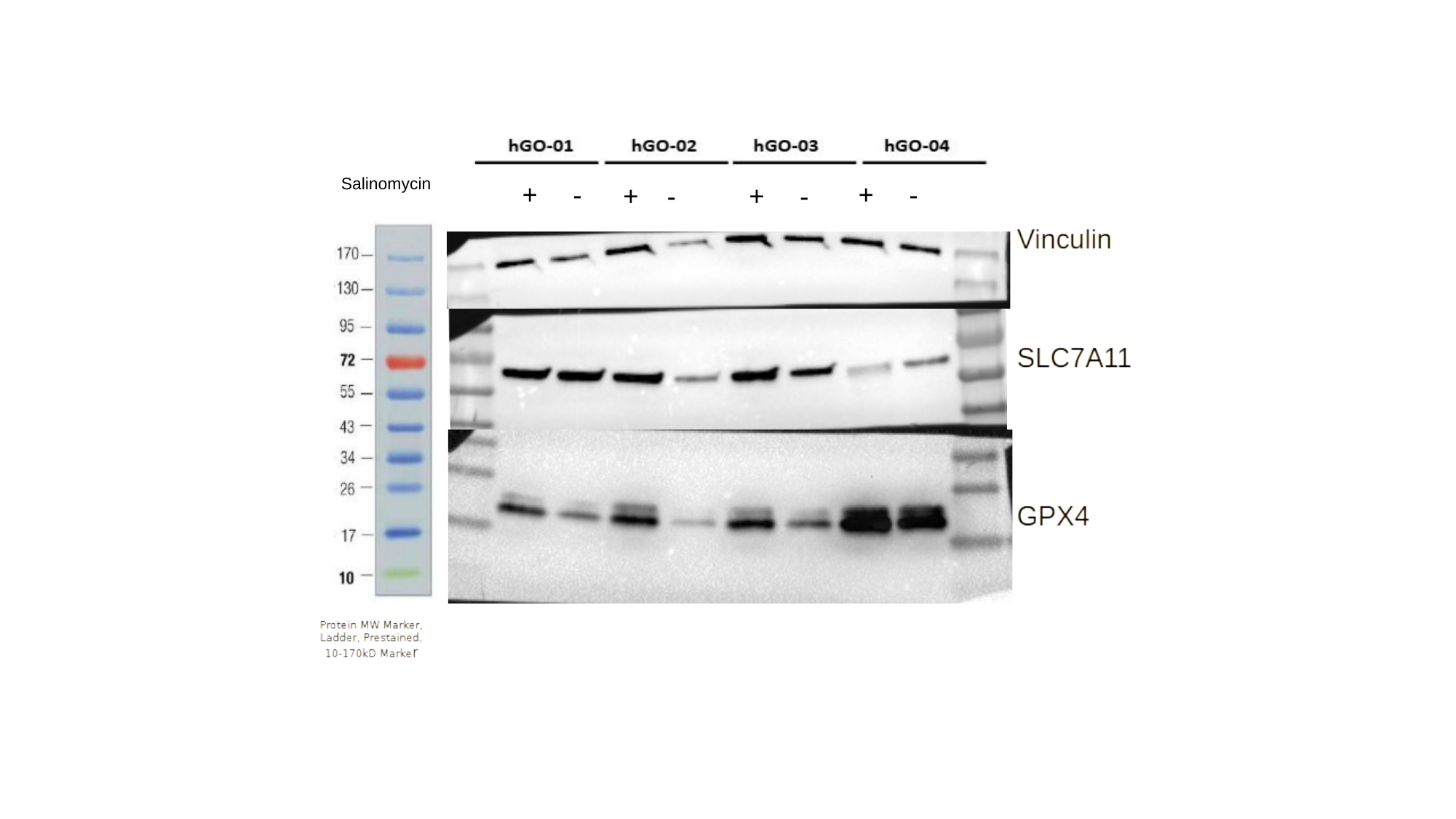

Salinomycin
+ -
+ -
+ -
+   -
