# Supplementary figures and images for: Salinomycin as a death switch: how gastric cancer cells choose their demise
Source: Cell Death Discov. 2026 Mar 24;12:171. doi: 10.1038/s41420-026-03058-2 (PMC13040004; doi:10.1038/s41420-026-03058-2)

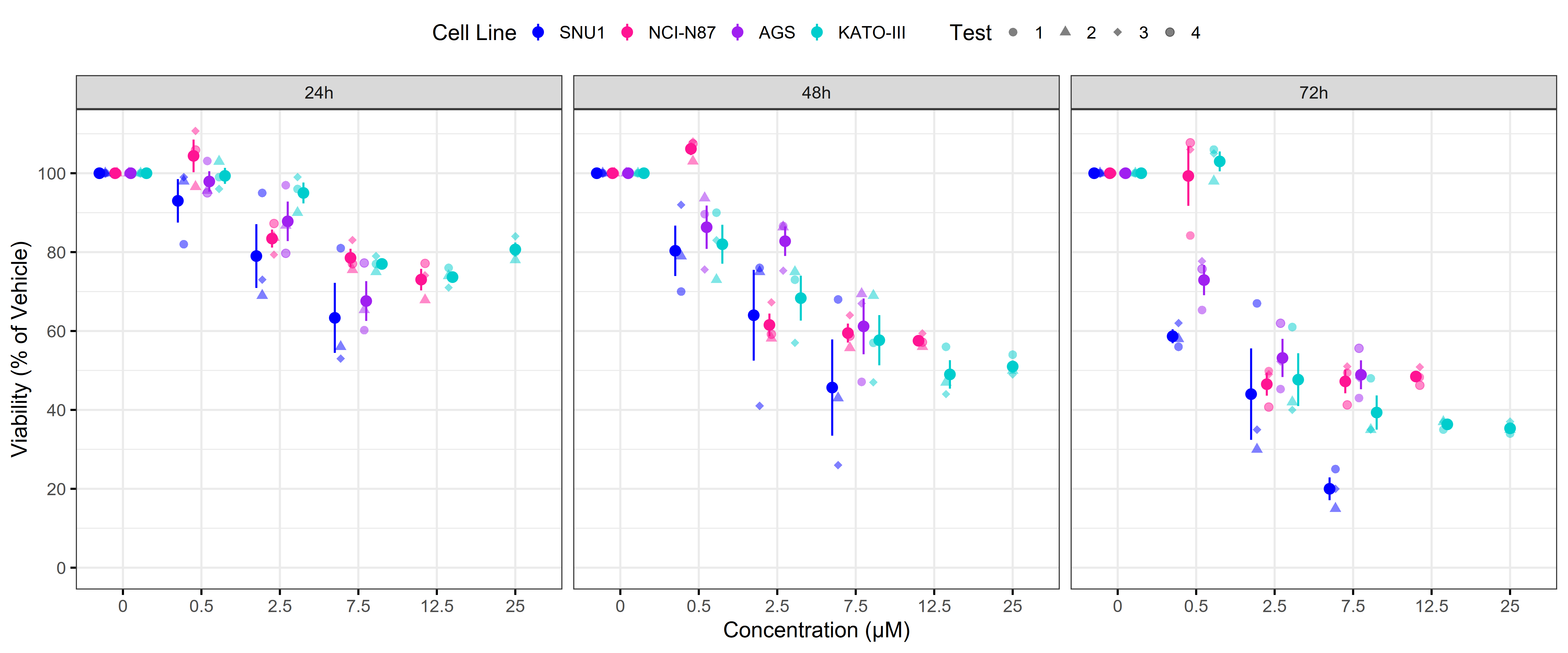

Supplement: Supplementary file 3 — Supplementary Figure 1 [file 41420_2026_3058_MOESM3_ESM.tif]
